# Knowledge domains and emerging trends of Genome-wide association studies in Alzheimer’s disease: A bibliometric analysis and visualization study from 2002 to 2022

**DOI:** 10.1371/journal.pone.0295008

**Published:** 2024-01-19

**Authors:** Fanjing Kong, Tianyu Wu, Jingyi Dai, Jie Cai, Zhenwei Zhai, Zhishan Zhu, Ying Xu, Tao Sun

**Affiliations:** 1 School of Intelligent Medicine, Chengdu University of Traditional Chinese Medicine, Chengdu, China; 2 School of Acupuncture-Moxibustion and Tuina, Chengdu University of Traditional Chinese Medicine, Chengdu, China; 3 Hospital of Chengdu University of Traditional Chinese Medicine, Chengdu, China; 4 State Key Laboratory of Southwestern Chinese Medicine Resources, School of Pharmacy, Chengdu University of Traditional Chinese Medicine, Chengdu, China; Dasman Diabetes Institute, KUWAIT

## Abstract

**Objectives:**

Alzheimer’s disease (AD) is a neurodegenerative disorder characterized by a progressive decline in cognitive and behavioral function. Studies have shown that genetic factors are one of the main causes of AD risk. genome-wide association study (GWAS), as a novel and effective tool for studying the genetic risk of diseases, has attracted attention from researchers in recent years and a large number of studies have been conducted. This study aims to summarize the literature on GWAS in AD by bibliometric methods, analyze the current status, research hotspots and future trends in this field.

**Methods:**

We retrieved articles on GWAS in AD published between 2002 and 2022 from Web of Science. CiteSpace and VOSviewer software were applied to analyze the articles for the number of articles published, countries/regions and institutions of publication, authors and cited authors, highly cited literature, and research hotspots.

**Results:**

We retrieved a total of 2,751 articles. The United States had the highest number of publications in this field, and Columbia University was the institution with the most published articles. The identification of AD-related susceptibility genes and their effects on AD is one of the current research hotspots. Numerous risk genes have been identified, among which *APOE*, *CLU*, *CD2AP*, *CD33*, *EPHA1*, *PICALM*, *CR1*, *ABCA7* and *TREM2* are the current genes of interest. In addition, risk prediction for AD and research on other related diseases are also popular research directions in this field.

**Conclusion:**

This study conducted a comprehensive analysis of GWAS in AD and identified the current research hotspots and research trends. In addition, we also pointed out the shortcomings of current research and suggested future research directions. This study can provide researchers with information about the knowledge structure and emerging trends in the field of GWAS in AD and provide guidance for future research.

## 1. Introduction

Alzheimer’s disease (AD) is a degenerative neurological condition characterized by a progressive decline in cognitive and behavioral functions, ultimately resulting in mortality [1]. As the most prevalent type of dementia, AD accounts for around 60–80% among all cases of dementia, emerging as one of the most costly, deadly, and burdensome diseases of this century [2, 3]. It has been more than 100 years since AD was first reported by Dr. Alois Alzheimer in 1906, yet it is still largely unknowable to us. Therefore, it is important to explore the underlying pathogenesis and identify causative and protective genes for us to better understand and treat AD. Studies of twins have demonstrated that genetic factors account for the major factors in the risk of AD [4]. Since the 1980s, *APP*, *PSEN1* and *PSEN2* have been found to be the cause of early-onset AD (EOAD), based on systematic linkage analysis [5]. However, EOAD accounts for only about 10% of the total number of AD, and it is late-onset AD (LOAD) that is the most common form of AD. Studies have shown that genes play an important role in the etiology of LOAD [6]. In 1993, ε4 of apolipoprotein E (AOPE) was found to be strongly associated with LOAD [7]. This groundbreaking discovery attracted our interest in genetic studies of AD. It was not until the advent of high-throughput genomic approaches, particularly genome-wide association studies (GWAS), that other risk factors associated with AD were gradually identified. GWAS, as an emerging tool to identify genetic risk factors associated with complex diseases, can identify single nucleotide polymorphisms (SNPs) more precisely by analyzing large amounts of genetic data to identify genetic locus variants associated with diseases [1]. Prior to the advent of GWAS, studies of AD were usually conducted using traditional methods such as family lineage studies, candidate gene studies, cellular and animal experimental studies, epidemiological studies, and neuroimaging studies. Although these methods can identify AD risk genes and pathogenesis to some extent, they are usually limited by the sample size, complex experimental design, and individualization bias, *etc*. GWAS enables the simultaneous assessment of the relationship between millions of genetic variants and AD, complementing the limitations in the sample size inherent to traditional methods and minimizing the occurrence of false-positive results to the greatest extent. Meanwhile, GWAS does not depend on specific biological samples or experimental conditions, thus it can reduce experimental bias and improve the accuracy of results. Particularly in the context of AD, GWAS excels in elucidating the disease’s multigenic effects and has facilitated the exploration of specific proteins and their roles in AD, providing insights into the underlying mechanisms of disease progression. The public availability of most GWAS-related databases and studies also fosters further research and collaboration in the field. As an effective method for genetic studies, GWAS has been widely used in the study of AD. In 2009, the first two large-scale GWAS results on AD were successively published, reporting the associations of *CLU*, *CR1*, and *PICALM* with the risk of AD [8, 9]. This groundbreaking achievement became a milestone in the field. Since then, a large number of GWAS for AD have been carried out and many novel risk loci for AD have been identified, such as *BIN1*, *TREM2*, *CD33*, *SORL1*, *CD2AP*, *ABCA7* and *EPHA1* [10].These loci have also been shown to be involved in multiple biological pathways in vivo, including immune actions, amyloid and tau protein processing, lipid metabolism, *etc* [8, 11–13]. The GWAS of AD reveals more genetic factors associated with AD. Furthermore, our understanding of the AD pathology can be enhanced by analyzing the biological functions of these genes, ultimately with a better guidance to AD prevention and treatment.

At present, a large number of studies on GWAS in AD have been conducted, generating amounts of scientific literatures, yet there is no study that has systematically summarized them. Bibliometrics is the study of academic publications, which assesses the basic characteristics and research hotspots of a field by quantitatively analyzing the relevant information of publications (including year of publication, journal of publication, author, country, keywords, *etc*.) [14]. Bibliometrics and visualization research methods have been widely used in various studies within the field of medicine. With the application of such methods, development trend in a particular disease, as well as in specific treatments or research methods for certain diseases, can be analyzed. CiteSpace and VOSviewer [15, 16], as two bibliometric software, can make statistical analysis and form a visual knowledge map based on publications. This study will use CiteSpace and VOSviewer to conduct a comprehensive analysis of GWAS in AD, in order to comprehensively summarize the development and evolution of the field, and to predict future research trends. It is anticipated that this study will help researchers gain a quick and comprehensive understanding of the field and provide certain ideas and directions for further research.

## 2. Materials and methods

### 2.1. Search strategy

The data for this research were obtained from the Web of Science Core Collection in the Web of Science (WOS), which includes science citation index expanded (SCI-EXPANDED) and social science citation index (SSCI). The search formula was as follows: ((((TS = (Alzheimer’s Diseases)) OR TS = (Alzheimer Disease)) OR TS = (Alzheimer Syndrome))) AND ((((TS = (genome-wide analysis)) OR TS = (genome-wide association study)) OR TS = (GWAS))). The search period is from 2002-01-01 to 2022-12-27.

### 2.2. Inclusion and exclusion criteria

Included articles are reviews and research articles on GWAS in AD; conference articles and articles that are not formally published will be excluded. The language will be limited to English. In addition, the included articles will be screened to exclude duplicate articles.

### 2.3. Bibliometric and visual analysis

Export the retrieved articles from WOS with full records and references. The target file would be exported in plain text format, named "download_X.txt". CiteSpace 6.1.R6 would be used for visual analysis of authors and co-cited authors collaboration networks, co-cited reference and research hotspots; VOSviewer 1.6.18 would be used for visual analysis of countries/regionals and institutions collaboration networks.

## 3. Result

### 3.1. Trends in the number of publications

**Fig 1:**
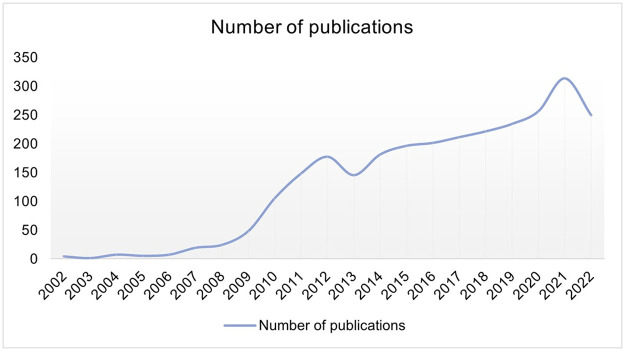
Annual publications of GWAS in AD from 2002 to 2022.

A total of 2,752 articles were retrieved from 2002 to 2022 (Fig 1), of which 2,217 were articles and 454 were reviews. Based on the trend of publication volume, it can be seen that the number of articles published on GWAS in AD has been increasing in the last 20 years. Among them, the number of published articles grew rapidly from 2009 to 2012, and reached the maximum number of published articles in 2021, which was 313 articles. Compared with 2021, the number of articles published in 2022 is slightly lower, which may be due to the fact that some articles could not be included because 2022 was not finished when this study was conducted.

### 3.2. Countries (regions) and institutions

**Fig 2:**
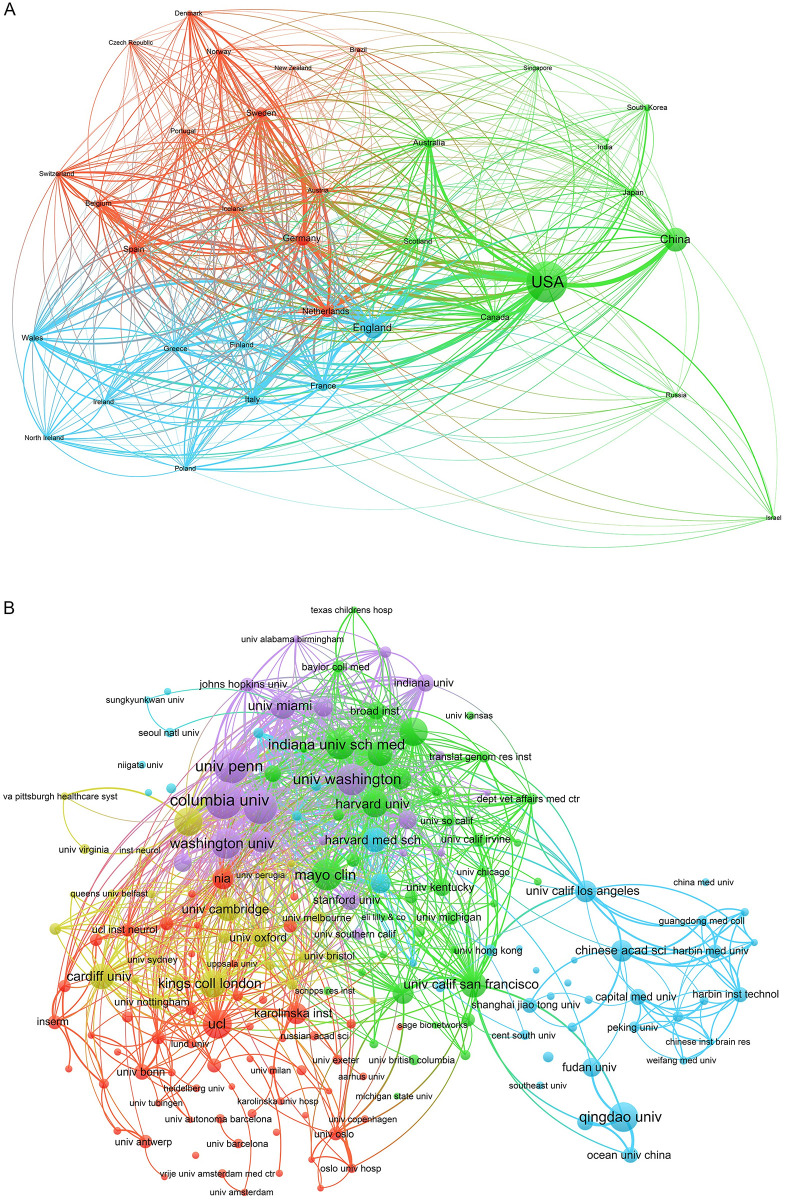
Collaborative networks between countries/regions and institutions. (A) Collaborative networks between countries/regions (B) Collaborative networks between institutions. The network diagrams are based on VOSviewer, and the node size indicates the number of publications; the link size refers to the cooperation Intensity.

During 2002 to 2022, GWAS in AD have been published in 85 countries and regions (Fig 2A). Table 1 lists the top 10 countries and regions with the most publications. According to the results, it is evident that the United States published the most studies in this field, which has arrived at 1459 articles in the 20-year period, accounting for 53% of total publications.

**Table 1:** Top 10 countries/regions contributing to publications on GWAS in AD.

Rank	Country/region	Counts	Citations	Total link strength
1	USA	1459	80685	1636
2	China	555	12712	344
3	England	454	40720	1287
4	Germany	237	22496	877
5	Netherlands	176	19427	774
6	Canada	160	18194	536
7	France	158	20161	758
8	Australia	146	10721	458
9	Italy	144	14632	576
10	Sweden	142	13982	615

Institutional collaborations (Fig 2B) involved a total of 2950 institutions, mainly dominated by universities and hospitals. Table 2 illustrated the top 10 institutions by publication counting, including universities and clinical institutions in the United States, the United Kingdom, and China. Among them, Institutions from the United States reached the highest number of published articles, accounting for 80%, and Columbia University is the institution that publishes the most publications. In the visualization map, different colors represent different clusters, and the lines represent inter-institutional collaboration. It indicates that close inter-institutional academic cooperation occurs in this field, and domestic cooperation is the main way. International cooperation should be strengthened in the future.

**Table 2:** Top 10 institutions contributing to publications on GWAS in AD.

Rank	Institutions	Country	Counts	Citations
1	Columbia University	USA	133	5618
2	University of Pennsylvania	USA	119	6206
3	Boston University	USA	117	5800
4	University of Washington	USA	106	6396
5	Mayo clinic	USA	102	6777
6	Washington University in St. Louis	USA	100	8124
7	Indiana University School of Medicine	USA	96	4513
8	Qingdao University	China	95	1805
9	University College London	UK	91	3800
10	University of Pittsburgh	USA	90	4236

### 3.3. Analysis of authors and co-cited authors

**Fig 3:**
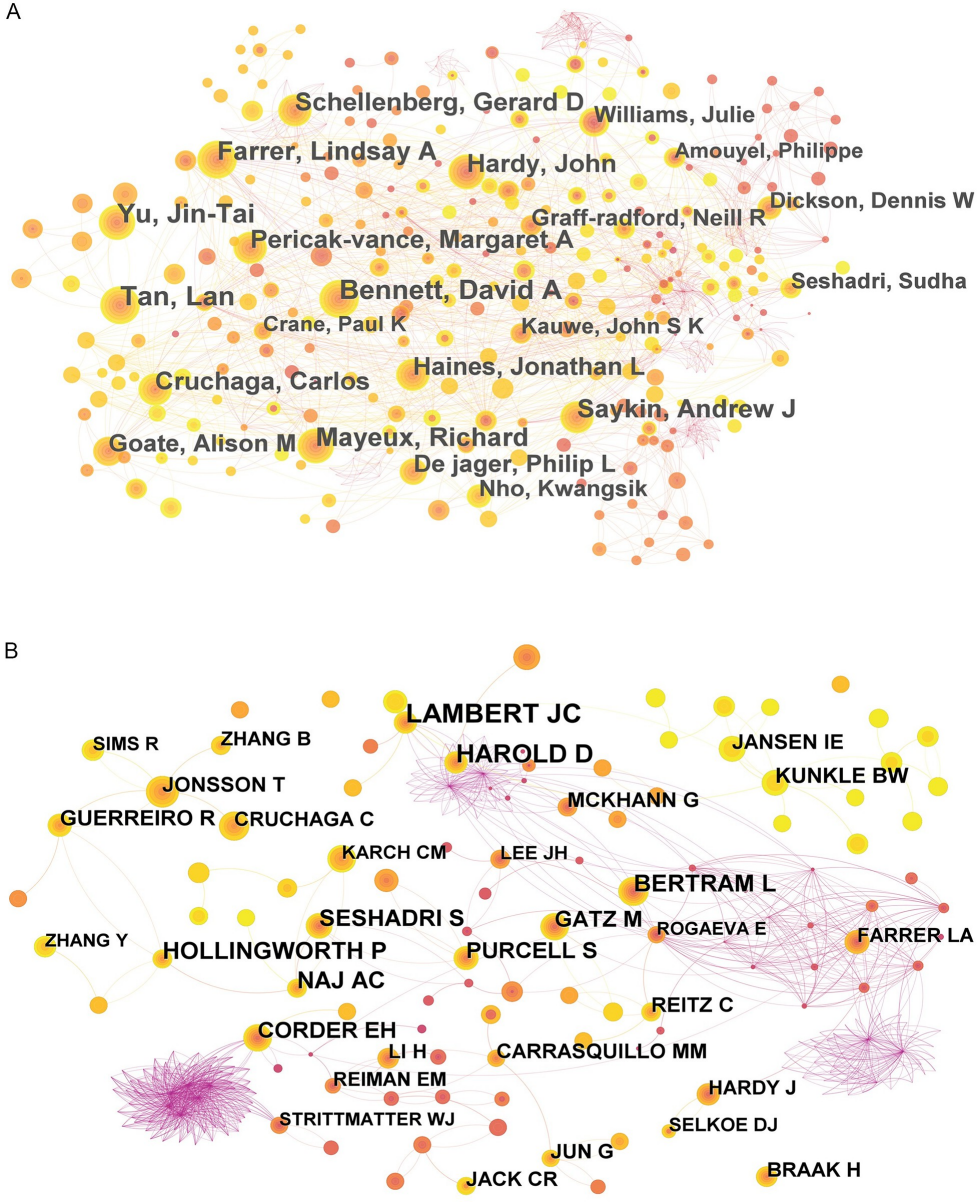
The knowledge map of active authors and co-cited authors on GWAS in AD. (A) Active authors. (B) Active co-cited authors.

The study of GWAS in AD was analyzed based on author’s collaboration network (Fig 3A) and author’s citation network (Fig 3B). Table 3 lists the top ten authors by the number of publications and citations, among which the top three authors with the most publications are Bennett, Tan and Yu, with 93, 91 and 90 publications, respectively. Lambert JC was the most cited author with 1463 citations, followed by Harold D with 990 citations. The remaining authors with a high number of citations had little difference in the number of citations.

**Table 3:** The top 10 authors and co-cited authors on GWAS in AD.

Rank	Author	Counts	Co-cited author	Counts
1	Bennett, David A	93	LAMBERT JC	1463
2	Tan, Lan	91	HAROLD D	990
3	Yu, Jin-Tai	90	NAJ AC	652
4	Mayeux, Richard	86	BERTRAM L	593
5	Saykin, Andrew J	80	HOLLINGWORTH P	593
6	Farrer, Lindsay A	75	SESHADRI S	549
7	Hardy, John	72	CORDER EH	435
8	Cruchaga, Carlos	71	PURCELL S	409
9	Pericak-vance, Margaret A	68	GATZ M	409
10	Schellenberg, Gerard D	64	GUERREIRO R	320

### 3.4. Co-cited reference analysis

**Fig 4:**
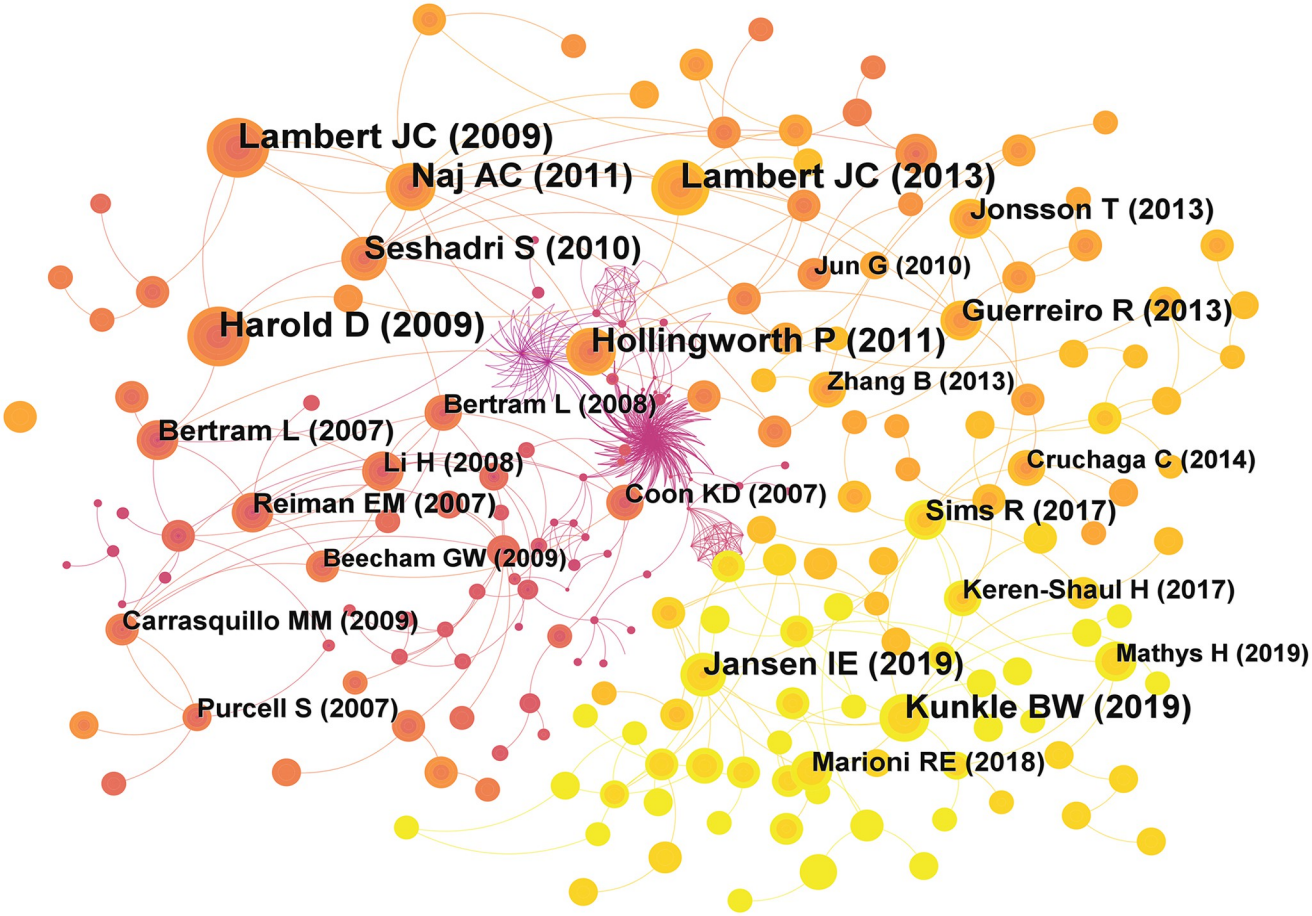
The knowledge map of co-citation reference on GWAS in AD.

The number of citations to an article represents the degree of attention to this topic. Namely, a higher number of citations means that the publication is more important. We used CiteSpace to visually analyze the references of the articles (Fig 4). The time slice was set to two years, and the top 30 most cited projects were selected from each slice. Nodes indicate the author and year of publication of the reference, the size of the node indicates the frequency of citation, the color of the node indicates different years, the connection between the nodes indicates that two references were cited at the same time, and the thickness of the line indicates the strength of the relationship. We list information of the top 10 most cited articles (Table 4). The articles are concentrated on three journals, Nature Genetics, JAMA and New England Journal of Medicine, of which a total of seven articles are from Nature Genetics. These 10 articles were all research articles which analyzed case group and control group through GWAS to find risk sites associated with AD. Harold D (2009) was the most frequently cited article, which identified the association of CLU and PICALM with AD by GWAS. In addition, Lambert JC (2013), Naj AC (2011), Hollingworth P (2011), Kunkle BW (2019), Jansen IE (2019), Seshadri S (2010) and Guerreiro R (2013) also combined the methods with meta-analysis.

**Table 4:** The top 10 cited references.

Rank	First author	Title	Year	Frequency	Journal	IF
1	Harold D	Genome-wide association study identifies variants at CLU and PICALM associated with Alzheimer’s disease	2009	448	NATURE GENETICS	41.307
2	Lambert JC	Genome-wide association study identifies variants at CLU and CR1 associated with Alzheimer’s disease	2009	425	NATURE GENETICS	41.307
3	Lambert JC	Meta-analysis of 74,046 individuals identifies 11 new susceptibility loci for Alzheimer’s disease	2013	417	NATURE GENETICS	41.307
4	Naj AC	Common variants at MS4A4/MS4A6E, CD2AP, CD33 and EPHA1 are associated with late-onset Alzheimer’s disease	2011	337	NATURE GENETICS	41.307
5	Hollingworth P	Common variants at ABCA7, MS4A6A/MS4A4E, EPHA1, CD33 and CD2AP are associated with Alzheimer’s disease	2011	329	NATURE GENETICS	41.307
6	Kunkle BW	Genetic meta-analysis of diagnosed Alzheimer’s disease identifies new risk loci and implicates Aβ, tau, immunity and lipid processing	2019	280	NATURE GENETICS	41.307
7	Jansen IE	Genome-wide meta-analysis identifies new loci and functional pathways influencing Alzheimer’s disease risk	2019	273	NATURE GENETICS	41.307
8	Seshadri S	Genome-wide analysis of genetic loci associated with Alzheimer disease	2010	259	JAMA	157.335
9	Guerreiro R	TREM2 variants in Alzheimer’s disease	2013	174	NEW ENGLAND JOURNAL OF MEDICINE	176.079
10	Jonsson T	Variant of TREM2 associated with the risk of Alzheimer’s disease	2013	146	NEW ENGLAND JOURNAL OF MEDICINE	176.079

### 3.5. Analysis of research hotspots

**Fig 5:**
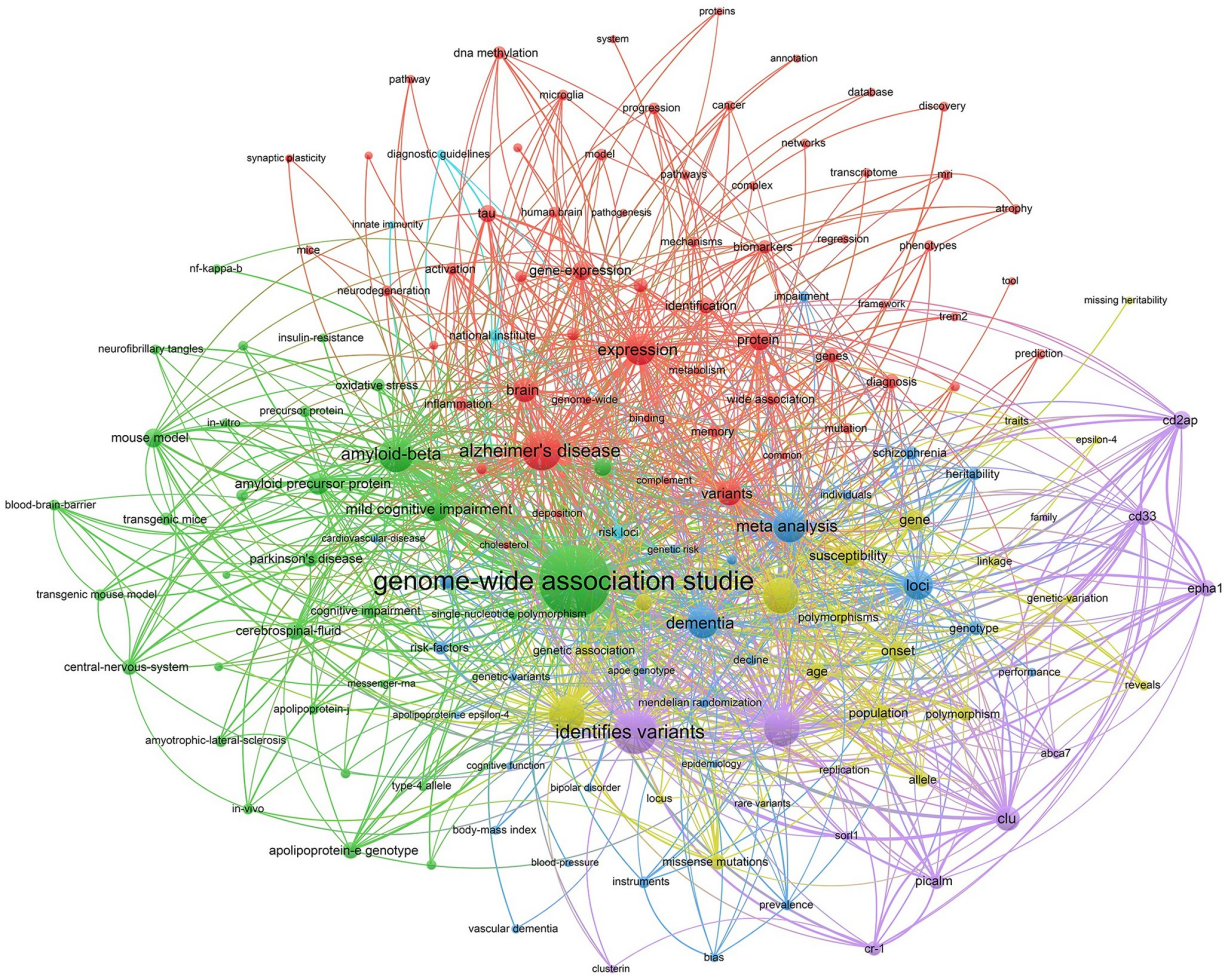
Keywords co-word network and clustering in the field of GWAS in AD.

As a label for an article, keywords can help readers quickly grasp the topic and content of the article. Analyzing the keywords of an article can give us a better understanding of the research hotspots and frontiers in the field [17]. In this study, a co-occurrence analysis and a cluster analysis (Fig 5) were performed on the keywords of the articles by using VOSviewer. After excluding the subject terms “genome-wide association studies” and “Alzheimer’s disease”, we summarized the top 10 keywords that appeared most frequently. As shown in Table 5, “identifies variants” was the most frequent words with 637 occurrences. Identifying variants is one of the core steps of GWAS, which refers to the process of discovering SNPs associated with specific features during the analysis of large amounts of genotype data. In addition, common keywords include risk, common variant, expression and loci, which are common steps and key concepts in the GWAS process. The *APOE* gene is located on chromosome 19 and has three alleles, APOE ε2, APOE ε3 and APOE ε4 [18]. Several studies [19–22] have demonstrated that *APOE* is the most strongly correlated genetic risk factor for LOAD, and it is a hot topic of current research, with a total of 435 occurrences in the keyword co-occurrence statistics. Meta-analysis as a research method has been often applied to GWAS, and by using meta-analysis, multiple independent GWAS results can be pooled to improve the reliability of the results and to better summarize the role of genetic variation in AD.

**Table 5:** The top 10 keywords on GWAS in AD.

Rank		Counts
1	Identifies variants	637
2	Risk	445
3	Common variants	440
4	APOE	435
5	Amyloid-beta	414
6	Meta-analysis	379
7	Expression	338
8	Dementia	326
9	Loci	299
10	Mild cognitive impairment	210

The results of the cluster analysis showed that these keywords were grouped into six categories. Table 6 summarizes the clusters and lists the keywords with higher frequency according to the frequency of occurrence. The first category is mainly concerned with the diagnostic, biomarker and molecular mechanisms of AD. Both phosphorylated tau protein and inflammatory response are typical pathological features and pathogenic hypotheses of AD, which are the hot topics of current research in AD pathology [23–25]. Gene expression variants are intimately associated to the pathogenesis and progression of AD, which can be identified by GWAS, and the corresponding diagnostic and therapeutic targets could be investigated based on the results. Biomarkers have great potential for the diagnosis and treatment of AD, meanwhile, protein and gene expression variants associated with abnormal tau protein deposition and inflammatory response are currently common research directions for biomarker studies [26–30]. The second category is mainly related to genetic factors of AD and related researches. It consists of identifying susceptibility loci for AD and analyzing the effect of *APOE* on AD by the application of GWAS. Cerebrospinal fluid contains a large number of molecules related to the nervous system, which often used as a research object in GWAS to find genetic risk factors related to AD [31, 32]. Aβ, produced when amyloid precursor protein (APP) is abnormally cleaved by β-secretase and γ-secretase, is one of the common pathological features and markers of AD [33]. When Aβ accumulates excessively it leads to cognitive impairment and the amyloid hypothesis is currently one of the most supported hypotheses for the pathogenesis of AD [24, 34]. The third category of keywords is mainly related to GWAS, including meta-analysis and Mendelian randomization (MR), which are common research methods in GWAS. MR is a method that uses genetic variation as a tool for causal inference [35]. MR, as a valuable tool in the field of GWAS, is often used in GWAS to assess the causal relationship between risk factors and certain manifestations of the disease [36]. The fourth category is similar to the third one, which is related to GWAS in AD, with keywords including risk, susceptibility, gene, onset, mutations, *etc*. The fifth category was mainly related to potential risk factors and genetic variants of AD. Most of the keywords were specific genes associated with potential risk of AD, including *CLU*, *CD2AP*, *CD33*, *EPHA1*, *PICALM*, *CR 1*, *ABCA7*, and *SORL1*. The sixth category had fewer keywords and more scattered topics, including risk loci, national institute, diagnostic guidelines and innate immunity.

**Table 6:** The clusters of keywords.

Cluster ID	
1	Alzheimer’s disease, expression, variants, brain, protein, gene-expression, tau, identification, diagnosis, biomarkers, inflammation
2	Genome-wide association studies, amyloid-beta, mild cognitive impairment, amyloid precursor protein, mouse model, susceptibility loci, cerebrospinal-fluid, apolipoprotein-e genotype
3	Meta-analysis, dementia, loci, genotype, cognitive decline, schizophrenia, risk-factors, heritability, instruments, mendelian randomization
4	Risk, susceptibility, gene, onset, mutations, age, population, missense mutations, polymorphism, allele
5	Identifies variants, common variants, clu, cd2ap, cd33, epha1, picalm, cr-1, abca7, sorl1
6	Risk loci, national institute, diagnostic guidelines, innate immunity

**Fig 6:**
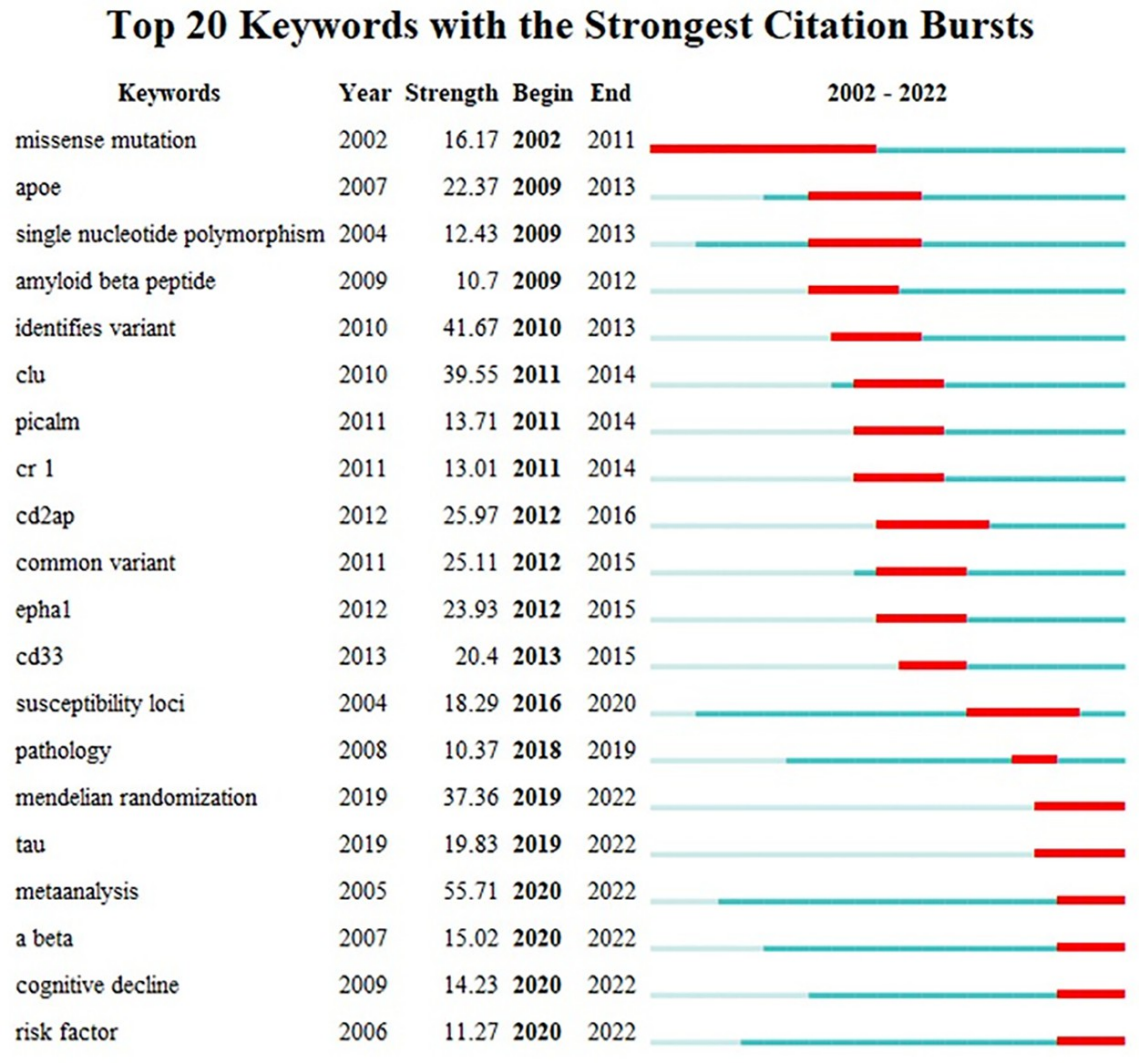
Keywords with bursts from 2002 to 2022.

With the development of this research field, its hotspots are also changing with time, and only analyzing keyword clustering has certain limitations which cannot reflect the changes of keywords over time. Keyword burst refers to a sudden increase in the frequency of keywords in a certain time period, meanwhile, analyzing keyword burst could provide assistance to understand the development trend of GWAS in AD research and identify research hotspots. In this study, we used CiteSpace for keyword burst analysis, and the top 20 burst keywords are listed in Fig 6. Missense mutation was the longest burst keyword, which lasted for almost 10 years. Meanwhile, we found that the keywords of burst before 2010 were mainly about the basic concepts of GWAS, and common pathological features and genetic risk genes of AD. It might be explained by the fact that the GWAS in AD field was just emerging that researchers mainly focused on the study of basic concepts. After 2010, researchers started to conduct a large number of GWAS-related studies of AD, and some new AD susceptibility genes started to appear in the outbreak keywords, indicating that researchers focused their research on the exploration of new risk genes. Mendelian randomization, tau, meta-analysis, Aβ, cognitive, and risk factor are the keywords that persist to date and are the hot directions of current research. In addition, mendelian randomization and tau were the most recent keywords among these words and became the focus of attention as soon as they appeared.

## 4. Discussion

This study conducted an analysis of the GWAS in AD using the method of bibliometrics. The article information was visualized and mapped using CiteSpace and VOSviewer, and a comprehensive analysis was performed based on the research results. Between 2002 and 2022, a total of 2752 articles were retrieved, of which 2217 were research articles, accounting for 81% of the total, and 454 were published in review. Currently many researchers conduct related studies, however, there are fewer review articles on this research and a systematic summary of the field is lacking. This may be due to the fact that GWAS in AD is a relatively new field, however, with the continuous research and a large number of new findings coming out, researchers are more inclined to produce original research papers to present the latest findings. Over the past 20 years, the number of publications has shown a consistent upward trend. The concept of GWAS can be traced back to 1996 [37], but it was not until a research article on GWAS published in Nature in 2005 [38] that it began to receive widespread attention and application. Therefore, only a few articles mentioned the concept of GWAS related to AD in the early days, and the number of related studies started to increase after 2005. The period from 2009 to 2012 was a period of rapid growth, representing the growing attention in this field, and since 2014, it has been in a steady growth state. Genetics research on AD has always been a highly interest research area. Previously, genetic research on AD was limited by sample size, frequency of specific variants and other factors, while SNPs at millions of loci could be analyzed by GWAS, allowing for higher resolution screening on the human genome to detect common diseases and disorders [39]. In addition, as an unbiased research method, it can exclude researchers’ subjective bias and discover more unknown gene-disease associations. Therefore, GWAS has attracted the attention of scholars in AD field, and a large number of related studies have been carried, and GWAS in AD has gradually become a popular research field. GWAS in AD have been published in 85 countries and regions, of which the United States having the highest number of publications and leading the field. The countries with the next highest number of publications are the United Kingdom and China. Due to factors such as geography and language, European countries tend to cooperate more frequently. Racial differences may have an impact on the study results, since the current study sample is mainly from Europe, and attention to other races should be strengthened in the future.

Analyzing the number of citations of articles can help us find the core research in the field. According to our survey, the top 10 most cited articles are all research articles, published mainly between 2009 and 2013. The most cited article was a study published in NATURE GENETICS in 2009 [8], which was the first large-scale GWAS in AD involving over 14,000 participants. This study detected an association between the *CLU* and *PICALM* genes and AD risk through GWAS. Another study, also published in 2009, was conducted by Lambert JC *et al*. [9]. In addition to the association of *CLU* with AD, they also reported an association of *CR1* with AD risk. In 2013 Lambert JC *et al*. performed a meta-analysis of data from the GWAS, the study population of European ancestry involving 74,046 individuals. This study identified four new susceptibility loci in addition to the analysis of known genes, providing strong evidence for the importance of APP and tau in AD pathology [40]. The remaining high-frequency cited articles all investigated AD risk genes through GWAS, including *MS4A4A*, *MS4A6A*, *TREM2*, *ABCA7*, *CD33*, *CD2AP*, *EPHA1*. In addition, we found two studies [12, 13] that were published less than three years but had a high number of citations that deserve readers’ attention. Jansen IE *et al*. [12] conducted a large-scale GWAS on AD and proxy AD of European ancestry, with the study being divided into three phases and encompassing over 400,000 participants. Similarly, Kunkle BW *et al*. [13] conducted a large-scale GWAS on LOAD, involving 94,437 individuals. Compared to the previous study, this research primarily focused on non-Hispanic white populations. In addition, both studies identified new risk loci, and the analyses revealed multiple pathogenic factors associated with AD, such as immune response, lipid metabolism, inflammation, Aβ, and tau proteins. It is noteworthy that both studies employed GWAS and meta-analysis methods, conducting large-scale research on AD patients and non-AD populations. This large-scale approach, with its excellent statistical validity and broad sample representation, ensured the stability, accuracy and reliability of the findings. This might be one of the reasons why these two studies have received substantial attention in a short period. Furthermore, the risk genes identified were thoroughly discussed, and the pathogenesis of AD was explored, which not only enhances the understanding of AD but also provides a basis for the pathogenesis and targeted treatment of AD.

Furthermore, the recently published articles were analyzed separately. It was found that recent publications have focused on GWAS and meta-analyses of multiracial populations as a way to validate previous findings and identify new genetic loci. Also, by continuing to analyze different races, it is possible to increase our understanding of the variability of AD genes across races. In addition, some of the current research focuses on in-depth studies of specific genes to enhance the understanding of the genetic and pathological mechanisms of AD. Recently, an article exploring the role of CD33 isoforms in microglia and AD has gained the attention of researchers after its publication.*CD33* is one of the high-risk genes associated with AD risk. It is suggested that *CD33* might influence the pathological process of AD by regulating the function of microglia. This article not only reviews *CD33*-related research, but also explores the potential possibility of targeting *CD33* for the treatment of AD.

With the increasing sophistication of GWAS research and computer technology, researchers are incorporating advanced techniques to further optimize GWAS studies. Yang *et al*., in their latest publication, introduced Causal Analysis using Regression Model Averaging (CARMA) to optimize fine-mapping in genome-wide meta-analyses, ameliorating the challenge of distinguishing between pathogenic and non-pathogenic variants in GWAS methods [41]. CARMA is a statistical method designed to help researchers gain a better understanding of the true causal relationships between variables. It robustly estimates causal effects in observational data, accurately identifying and addressing potential confounding issues. LD information in previous studies is usually obtained from external reference panels. However, this may lead to inconsistencies between the LD information and the GWAS summary statistics, resulting in biased mapping results. CARMA, a Bayesian model, not only explains the discrepancies between the summary statistics and the LD from the reference panels, but also integrates the data with the functional annotations, which improves the GWAS results’ accuracy [41]. In recent years, the use of machine learning (ML) to detect and classify diseases has attracted a lot of attention, and more researchers are beginning to use ML to address complex diseases such as AD [42]. ML, especially deep learning, is considered a powerful tool for GWAS data analysis due to its ability to process large-scale data, automatically extract key features, and identify complex interaction effects between multiple SNPs [43]. A study employed a convolutional neural network model combined with principal component analysis to process MRI data from brain regions. The extracted feature vectors were then used as endophenotypes in GWAS to identify genetic variants linked to AD. This automated feature extraction approach offers more comprehensive information compared to traditional methods, enhancing the accuracy of predicting genetic risk genes. Furthermore, integrating brain imaging data with GWAS offers new insights into the genetic and biological mechanisms of AD.

In this study, keywords were analyzed to find out the trends and research hotspots in the field. A total of 8566 keywords and six clusters were obtained. Comprehensive analysis of the keyword and keyword burst results indicated that GWAS in AD mainly focuses on discovering susceptibility genes and studying their effects on AD. More than 100 AD-associated risk loci have been identified in existing studies. In this study, the hot genes currently under investigation were identified based on keyword analysis (Table 7). As one of the most significant genetic risk factors of AD, *APOE* has always been a focus of research [44]. APOE is a protein consisting of 299 amino acids, primarily involved in the transport and metabolism of cholesterol. It is believed to play a vital role in the brain, particularly in lipid transport and damage repair. APOE assists in the transport and metabolism of lipids, such as cholesterol, by binding to lipid molecules and forming lipoprotein particles [20, 45]. GWAS have confirmed the APOE *ε4* allele as a major genetic risk factor of AD, and in particular, it is strongly associated with the risk of LOAD [8, 9]. The effect of APOE4 on AD is thought to implicate multiple pathological manifestations. It was shown that APOE can promote the metabolism and clearance of Aβ, while the variation of the APOE4 gene would affect the ability of APOE to bind Aβ, thereby disrupting the metabolism and clearance of Aβ, leading to abnormal accumulation and deposition of Aβ and increasing the risk of AD [46]. In addition, APOE4 can worsen neurodegeneration that is mediated by tau and impact the pathology of tau proteins [47–49]. APOE4 has also been found to cause metabolic dysregulation in astrocytes and microglia, leading to inflammation, neuronal damage and other AD pathologies [50]. In addition, APOE4 can impair myelin formation in the brain by interrupting astrocyte-derived lipid transport, thereby disrupting neural signaling and leading to cognitive and motor deficits in AD patients [51]. APOE4 has been found to be expressed in various ethnic groups, including African descent, Caucasian, Hispanic, Asian, *etc*., with a higher frequency of expression in African and Caucasian [52]. The effects of APOE4 on different races are still unclear, and a number of experiments have been conducted to explore. For instance, one study involving African, Caucasian, and Latino populations revealed that APOE4 was associated with poorer performance in episodic memory, particularly among Caucasians [53]. Conversely, another study encompassing Hispanics, African Americans, Hispanics, and non-Hispanic white Americans indicated that the influence of APOE4 on AD did not exhibit significant racial variability [54]. Although APOE4 carriers show a higher prevalence in all races, the extent to which there is racial variability in the impact of AD deserves further study in the future. At the same time, researchers are also currently focusing on whether APOE4 has gender-differentiated effects on AD. It has been found that APOE4 has a more significant effect on women in terms of cognition and language [55]. Individuals carrying APOE4 in females experienced a faster decline in memory capacity than males, and in addition, APOE4 showed a stronger association with tau pathology in females [56–59].

**Table 7:** Locus validated for association with AD risk by GWAS.

Locus	Chr	Biological functions	SNP	Alleles	Impact on AD risk	GWAS
*APOE*	19	Lipid metabolism	rs429358; rs405509	T/C; T/G	↑; ↓	[8, 112–115]
*CLU*	8	Cell apoptosis, immune response, inflammation response	rs9331896; rs2279590; rs9331888; rs1532278	C/T; T/C; C/G; T/C	↓; ↓; ↑; ↓	[8, 86, 115]
*CD2AP*	6	Endocytosis	rs9349407; rs9473117	G/C; A/C	↑; ↑	[83, 86]
*CD33*	19	immune response, inflammation response, signal transduction	rs3865444; rs3826656	C/A; G/A	↓; ↑	[83, 86]
*EPHA1*	7	Neuronal development, signal transduction, inflammation response	rs10808026; rs11767557	C/A; T/C	↓; ↓	[83, 86]
*PICALM*	11	Neuronal development, endocytosis	rs3851179; rs561655	T/C; G/A	↓; ↓	[8, 86, 115, 116]
*CR1*	1	immune response, inflammation response	rs3818361; rs4844610	A/C; A/C	↑; ↑	[83, 86]
*ABCA7*	19	Lipid metabolism, inflammation response	rs3764650; rs3752246	T/G; G/C	↑; ↑	[83, 86]
*TREM2*	6	immune response, inflammation response	rs75932628; rs143332484	C/T; C/T	↑; ↑	[117]

Chr: chromosome

*CLU* is another major brain apolipoprotein gene after *APOE*, which is widely expressed in the central nervous system [60]. Similar to *APOE*, *CLU* is synthesized and released by astrocytes and neurons, playing a role in lipid metabolism within the brain [61]. Harold *et al*. [8] and Lambert *et al*. [9] discovered through GWAS that this gene is associated with the risk of AD. Studies have shown that the level of CLU will increase when the brain is damaged or chronically inflamed. Animal experiments have shown that mice deficient in *CLU* are more susceptible to Aβ neurotoxicity. It has also been suggested that *CLU* may be beneficial for the clearance of Aβ in vivo [62, 63]. In addition, *CLU* has been shown to cooperate with *APOE* in inhibiting Aβ deposition [62]. Depending on the SNP variation, the *CLU* gene forms several different alleles, which affect AD to varying degrees. Case-control studies in Chinese population have demonstrated the effect of *CLU* on AD susceptibility [64–67]. Notably, the T allele of rs11136000 and the A allele of rs2279590 showed significant protective effects [64]. The T allele of rs11136000 was found to be associated with better cognitive performance in older adults [68, 69]. The role of rs11136000 has also been confirmed in meta-analyses involving both Caucasian and Asian populations [65, 70, 71]. In Caucasian populations, *CLU* rs93331888 and *CLU* rs11136000 were found to be associated with AD [8, 9]. However, no significant association was observed in studies involving Asian populations [72, 73]. This suggests a notable racial variation in the impact of *CLU* on AD, highlighting the need for further research through large-scale experiments in the future.

At the same time, Lambert *et al*. [9] also found that the *CR1* gene could participate in Aβ clearance together with *APOE*. The *CR1* gene, located on chromosome 1, serve as the primary receptor for complement proteins and can influence AD pathology by regulating complement protein activity. GWAS has revealed that *CR1* shows a strong correlation with LOAD risk. Studies suggest that *CR1* primarily affects the clearance of Aβ by participating in the body’s immune system [74]. Various SNPs of *CR1*, including rs11118322, rs17259045, rs12567945, rs1323721, *etc*., have been found to affect Aβ levels in the brain to different extents, among which rs12567945 has an inhibitory effect on Aβ. Additionally, *CR1* rs6656401 and rs4844609 were also found to be associated with intelligence level, which may affect cognitive function in AD [9, 75, 76].

*CD2AP* is a protein-coding gene whose product is thought to be involved in the regulation of receptor-mediated endocytosis and the immune system [77]. GWAS found that *CD2AP* is associated with tau protein toxicity, thereby affecting the occurrence of AD [13]. This result was also confirmed in the Drosophila AD model [78]. A cohort study has found that *CD2AP* rs9296559 is associated with higher tau levels in cerebrospinal fluid [79]. In addition, different SNPs of the *CD2AP* gene are believed to affect Aβ levels; for instance, *CD2AP* rs9349407 was found to cause an increase in plaque load [80]. Animal studies have found that *CD2AP* also leads to an increase in Aβ and the Aβ42/Aβ40 ratio [81]. *CD2AP* is also thought to potentially have an indirect effect on AD by interacting with other genes or influencing cardiovascular and other risk factors [77]. Numerous studies have been conducted on different races to investigate the effects of *CD2AP* on AD and racial variability. However, the results of current studies remain controversial. A study of *CD2AP* rs9349407 concluded that studies in Asians, Americans, and Europeans may be negative due to factors such as small sample size. A meta-analysis of this study by expanding the sample size showed that *CD2AP* rs9349407 was associated with AD susceptibility [82]. More large-scale studies should be conducted in the future to help us better understand the relationship between the *CD2AP* gene and AD in different ethnic backgrounds.

The *CD33* gene encodes a cell surface receptor protein, the primary function of which involves immune regulation and cell signaling [83]. Studies have been conducted on multiple SNPs of *CD33*. One GWAS identified a correlation between *CD33* and AD, reporting that rs3826656 affects the development of late-onset AD [84]. Subsequently, numerous GWAS have been conducted to determine the association of other SNPs of *CD33* with susceptibility to AD, including rs3865444, rs12459419, rs2455069, *etc* [83, 85–88]. Different polymorphisms of *CD33* are thought to influence the AD pathological process by affecting microglial activation, interfering with microglial-mediated Aβ clearance, and promoting the accumulation of senile plaques [89–91]. *EPHA1* is a membrane-bound protein that is mainly involved in synaptic development and plasticity [92]. In addition, it is also thought to play a role in inflammation and apoptosis [93, 94]. A GWAS study found that the non-coding form of *EPHA1* showed an association with AD [95]. Polymorphisms in *EPHA1* may influence the pathogenesis of AD by affecting the production and clearance of Aβ [91]. In addition, the GWAS study reported that the rs11767557 variant of the *EPHA1* gene appeared to be more highly correlated with susceptibility to AD in European populations, particularly with LOAD [95]. *PICALM* is widely expressed in all cells, with significant expression in neurons. It is considered to have a crucial role in cellular endocytosis, neuronal development, and synaptic plasticity. *PICALM* could affect the processing of APP through the endocytic pathway, which ultimately results in alterations in Aβ levels [8]. In addition, *PICALM* is thought to be involved in the regulation of tau proteins and to influence tau pathology. *PICALM* expression has been found to be dysregulated in the brains of LOAD patients, as evidenced by increased immunoreactivity in microglia and reduced protein levels in microvessels [96, 97]. Different loci of *PICALM* have been studied for AD risk in various races. The effects of different loci of *PICALM* on AD exhibit some racial variability, with a stronger correlation to AD risk observed in Caucasians. This may be due to the fact that more experiments are currently being conducted on Caucasians, and attention to other races should be increased in the future. For example, *PICALM* rs592297 demonstrated a risk for AD in Caucasians; however, it did not seem to have an effect on Asians [98]. There are still limitations, such as small sample sizes, in the current research on other races, so that large-scale experiments should be further conducted to study other races to improve the accuracy of the conclusions.

*ABCA7* belongs to the family of transporter proteins and is predominantly expressed in microglia and neurons [99]. It suggest that *ABCA7* is mainly involved in cell phagocytosis and lipid regulation [99, 100]. *ABCA7* is thought to be an important risk gene for LOAD [101]. The exact mechanism by which *ABCA7* functions remains unclear, but it may affect AD by regulating the ability to transfer phospholipids to lipoproteins, such as *APOE* and *CLU* [102, 103]. Additionally, *ABCA7* may also impact AD by regulating the phagocytic activity and APP processing in microglia [100, 104]. Studies have also revealed that mutations in the *ABCA7* gene lead to increased Aβ production and neuroinflammatory plaques *in vivo* [80, 104]. *ABCA7* has been identified as a risk gene for AD across multiple ethnicities, with certain polymorphisms demonstrating notable racial predispositions, such as rs115550680 in African Americans [105], rs3764648, rs3752229, rs150594667, and rs4147914 in Asians [106], and rs3764650 in Caucasians [107]. *ABCA7* is believed to exert a more potent impact on individuals of Africans and is considered to have a stronger effect on African Americans compared to *APOE* [105]. Moreover, different alleles of *ABCA7* appear to exhibit gender variability in their impact on AD. Research has found that the *ABCA7* SNP (rs3764650) is significantly associated with cognitive impairment exclusively in women, while *ABCA7* rs3764650 is only related to cognitive dysfunction in men [108]. Such discrepancies may be attributed to factors like sample size and study population, deserving further in-depth investigation in the future. *TREM2* is a protein primarily expressed on the surface of immune system cells, with the most significant expression in microglia cells in the brain [109]. *TREM2* plays a vital role in the phagocytosis of amyloid plaques by microglia cells, and mutations in the *TREM2* gene may interfere with microglial function, leading to an increased risk of AD [110, 111]. The susceptible loci that have been identified affect AD mainly through various functions including APP processing, lipid metabolism, endocytosis, Aβ and tau accumulation, and immune regulation. Microglia in the brain are believed to play a crucial role in immune regulation and thus warrant the attention of researchers. It is important to note that this study only highlights a few risk genes that are currently being extensively researched. In recent years, additional candidate loci such as *BIN1*, *SORL1*, *MS4A*, *SPI1*, *TOMM40*, *etc*. have been identified through GWAS, and these genes also deserve continued attention and further research in the future.

Based on the analysis results of GWAS-related articles in this study as well as data from other gene databases, the top 10 biological, molecular, and cellular pathways most related to AD were identified. They are the metabolism of Aβ protein, the phosphorylation and aggregation of tau protein, the neuroinflammatory response, cholesterol metabolism and transport, cell apoptosis and survival pathways, neuronal signal transduction, oxidative stress response, organization and stability of the cytoskeleton, synthesis and release of neurotransmitters, and neural growth and repair. Aβ and phosphorylated tau are two hallmark pathological manifestations of AD [3]. The metabolism of Aβ encompasses its production, processing, and degradation. Aβ is produced through the aberrant cleavage of APP, and when APP is abnormally cleaved by β-secretase and γ-secretase, it results in the formation of an Aβ fragment [118]. A variety of genes have been found to be involved in Aβ metabolism. *APOE* is thought to affect Aβ metabolism in an isoform-specific manner. *APOE4* is thought to significantly impair Aβ clearance, while *APOE2* only slightly inhibits it [119]. In addition, genes such as *BACE1*, *CR1*, and *ABCA1* have been found to affect AD by influencing the Aβ metabolic pathway [120, 121]. The accumulation of Aβ may interfere with the synthesis and release of neurotransmitters, leading to impaired communication between neurons [122–124]. At the same time, nerve injury may also affect the release of nerve growth factor, further affecting neuronal growth and repair. Tau protein is a major component of neurofibrillary tangles, and aberrant phosphorylation of tau protein may lead to its aggregation to form neurofibrillary tangles, affecting AD pathology [125]. In addition, abnormal aggregation of tau proteins may disrupt the cytoskeleton of neurons, leading to morphological and functional changes in neurons. *APOE* has been found to directly affect tau proteins [49]. In addition, genes such as *CR1*, *SORL1*, and *ABCA7* have also been identified to influence AD by affecting the phosphorylation and aggregation of tau protein [126, 127]. Neuroinflammation is thought to play a crucial role in the progression of AD. Accumulation of Aβ may activate microglia and astrocytes, leading to an inflammatory response that accelerates neuronal damage [128]. APOE4 has been found to increase the inflammatory response, while the presence of inflammation exacerbates symptoms in patients with *APOE4* [129]. *CLU*, *CR1*, *TREM2*, and *CD33* have also been identified to involve in the AD inflammatory response [130, 131]. Cholesterol plays a crucial role in the structure and function of neuronal membranes [132]. *APOE4*, *CLU* and *ABCA7* were found to involve in cholesterol metabolism [133]. Abnormal cholesterol metabolism and transport may affect Aβ production and clearance, further accelerating the progression of AD [134]. Cell apoptosis is a biological mechanism that can lead to AD when excessive apoptosis occurs [135]. Meanwhile, neuronal death in brain regions, induced by apoptosis, can result in cognitive deficits [136]. *APOE4* has been found to induce apoptosis and synaptic dysfunction, thus aggravating AD. Additionally, *CLU* knockout has been observed to cause apoptosis in AD cells [137]. The accumulation of Aβ might interfere with inter-neuronal signaling, leading to cognitive decline. Oxidative stress is considered a key factor in AD to lead to neuronal damage [138]. Multiple genes, such as *APOE4* and *CLU*, have been found to be associated with the oxidative stress response in AD [139–141].

In addition to studying susceptibility genes and their biological mechanisms, risk prediction for AD is also a hot research interest in the field. Risk prediction research is mainly based on genetic risk factors or biomarkers to establish risk assessment models, as well as establishing risk assessment models through neuroimaging analysis. Established risk assessment models based on biomarkers generally involves detecting AD-related biomarkers (such as tau protein and Aβ protein in cerebrospinal fluid) and combining them with individual age, gender, family history, and other factors for comprehensive evaluation. In addition, because of the similar or partially overlapping pathological mechanisms with AD, researchers have also attempted to investigate whether the AD-associated genes that have been identified are also involved in other neurodegenerative diseases, such as Parkinson’s disease and amyotrophic lateral sclerosis.

Given the close association between the diagnosis, prevention, and treatment of AD, a comprehensive discussion on these keywords was conducted, aiming to enhance a holistic understanding of AD and elevate its value in clinical practice. Since there is no ideal drug that can completely cure AD, it is crucial to diagnose AD in a timely and accurate manner. MCI is an early clinical manifestation of AD, and patients at this stage experience mild impairment of memory and behavior. Early detection and diagnosis help patients to receive timely treatment, thus slowing down the progression of the disease. When AD progresses to an advanced stage, patients will experience severe cognitive impairment, eventually leading to dementia. A timely diagnosis of dementia not only helps doctors to develop a personalized treatment plan for the patient, but also provides the necessary care and emotional support to improve the patient’s quality of life. LOAD is thought to be influenced by a combination of genes and the environment, and gene-environment interactions (GxE) may accelerate or exacerbate cognitive impairment [142]. Currently GxE has received attention from researchers and a series of studies have been carried out to help better prevent AD. For example, cadmium (Cd), a toxic heavy metal, is mainly exposed to humans through diet (*e*.*g*., oysters, peanuts, animal offal, *etc*.) and smoking [143]. Long-term exposure to Cd can damage organs such as the liver and kidneys. Moreover, Cd can cross the blood-brain barrier, leading to inflammation, neuronal apoptosis, and other pathologies that contribute to cognitive decline [144, 145]. Additionally, Cd is believed to exacerbate cognitive impairment in the presence of *APOE4* [146]. This phenomenon has also been observed in studies of another heavy metal, lead (Pb) [147]. Therefore, it’s reasonable to assume that chronic exposure to heavy metals might intensify or accelerate cognitive decline in individuals with *APOE4* [146]. Research suggests that the Mediterranean diet helps reduce the risk of AD and dementia, offering an effective approach for the prevention and treatment of AD [148, 149]. This effect is particularly pronounced in individuals who do not carry the *APOE4* allele [150].

Currently, tools for diagnosing AD include psychological assessment, imaging and laboratory tests. Biomarkers can reflect the pathophysiological characteristics of AD by detecting specific substances in biological fluids [151]. These markers can directly show the biological changes of the disease, which is valuable for early diagnosis of AD and dynamic monitoring of disease progression. Currently, the main biological tests are MRI, PET scanning and cerebrospinal fluid testing [152]. Among these, cerebrospinal fluid is the most commonly used method to assess AD progression, primarily by detecting Aβ42 or Aβ42:40 ratios, as well as total tau and phosphorylated tau levels [152]. In recent years, intensive research on genetic risk and pathological features of AD has provided new directions for the prevention and treatment of AD. Genes associated with AD identified through GWAS have attracted extensive attention from researchers. These genes play a crucial role in the onset and progression of AD, and targeted therapy against these genes may pave new avenues for the treatment of AD.

Based on the results of our analysis, the field is currently in a rapidly developing period and a large number of studies have been conducted. We note that current research mainly focuses on Caucasians with relatively few studies conducted on other races, which means that we may miss some key genetic variants in other populations. Due to genetic differences between races, genetic variations commonly found in Caucasians may be rare or non-existent in other races. The rs9331888 polymorphism of the *CLU* gene was found to affect AD risk in Caucasians, but there was no significant association in East Asian populations [73]. *CR1* rs6656401 is thought to have an impact on AD risk in Europeans, however whether it has an effect on other ethnic groups is controversial [153]. Some scholars have suggested that the negative results of rs6656401 in East Asian populations may be related to the small sample size, and the association of *CR1* rs6656401 with AD was confirmed by meta-analysis [154]. Therefore, we suggest that more GWAS studies with large samples and multiple races should be conducted in the future to understand more about the genetic heterogeneity of AD in different populations. GWAS can identify risk loci associated with AD which help to explore the pathogenesis of AD and thus provide a theoretical basis for the next step in the treatment and prevention of AD. However, there is still a long way to go from genetic studies to clinical translation. In addition to determining the relevance of genes and AD, how these genes function in the pathogenesis of AD is the focus of research. Furthermore, a large number of experimental studies should be conducted to validate the results. In parallel, our findings on risk genes may guide the design of animal models that better reflect the characteristics of human AD.

Meanwhile, this study has certain limitations. Due to the limitation of the analysis software only articles in WOS were included in this study, and some articles from other databases may have been omitted. In addition, the language of this study was limited to English, and articles published in other languages were not included. At the same time, due to the short publication time of some studies, it will have an impact on the citation frequency statistics, resulting in the underestimation of some recently published articles, which will have a certain impact on the research results.

This study explored the GWAS in AD from 2002 to 2022 using CiteSpace and VOSviewer software. A comprehensive summary of the GWAS field of AD was provided by analyzing the number of publications, countries/regions and institutions of publication, authors and cited authors, highly citations, and research hotspots. This study could facilitate relevant researchers to gain insight into the developmental trends and hotspots in this field and further comprehend the genetic risk factors and mechanisms of AD. Moreover, this study also points out the shortcomings of the current study and the direction of future research, providing a valuable reference for researchers. Future GWAS in AD should be strengthened in terms of sample size, ethnic diversity, and experimental validation. Continued research into the genetic risk and mechanisms of AD and the development of targeted therapeutic in response to the findings are major focus of future research and urgently need to be tackled.

## References

[1] Shen L, Jia J. An Overview of Genome-Wide Association Studies in Alzheimer’s Disease. Neurosci Bull. 2016;32: 183–190. doi: 10.1007/s12264-016-0011-3 26810783 PMC5563735

[2] Jung YJ, Kim YH, Bhalla M, Lee SB, Seo J. Genomics: New Light on Alzheimer’s Disease Research. Int J Mol Sci. 2018;19: 3771. doi: 10.3390/ijms19123771 30486438 PMC6321384

[3] Scheltens P, De Strooper B, Kivipelto M, Holstege H, Chételat G, Teunissen CE, et al. Alzheimer’s disease. Lancet. 2021;397: 1577–1590. doi: 10.1016/S0140-6736(20)32205-4 33667416 PMC8354300

[4] Gatz M, Reynolds CA, Fratiglioni L, Johansson B, Mortimer JA, Berg S, et al. Role of genes and environments for explaining Alzheimer disease. Arch Gen Psychiatry. 2006;63: 168–174. doi: 10.1001/archpsyc.63.2.168 16461860

[5] Bellenguez C, Grenier-Boley B, Lambert J-C. Genetics of Alzheimer’s disease: where we are, and where we are going. Curr Opin Neurobiol. 2020;61: 40–48. doi: 10.1016/j.conb.2019.11.024 31863938

[6] Tanzi RE. A genetic dichotomy model for the inheritance of Alzheimer’s disease and common age-related disorders. J Clin Invest. 1999;104: 1175–1179. doi: 10.1172/JCI8593 10545516 PMC409831

[7] Strittmatter WJ, Saunders AM, Schmechel D, Pericak-Vance M, Enghild J, Salvesen GS, et al. Apolipoprotein E: high-avidity binding to beta-amyloid and increased frequency of type 4 allele in late-onset familial Alzheimer disease. Proc Natl Acad Sci U S A. 1993;90: 1977–1981. doi: 10.1073/pnas.90.5.1977 8446617 PMC46003

[8] Harold D, Abraham R, Hollingworth P, Sims R, Gerrish A, Hamshere ML, et al. Genome-wide association study identifies variants at CLU and PICALM associated with Alzheimer’s disease. Nat Genet. 2009;41: 1088–1093. doi: 10.1038/ng.440 19734902 PMC2845877

[9] Lambert J-C, Heath S, Even G, Campion D, Sleegers K, Hiltunen M, et al. Genome-wide association study identifies variants at CLU and CR1 associated with Alzheimer’s disease. Nat Genet. 2009;41: 1094–1099. doi: 10.1038/ng.439 19734903

[10] Li Y, Laws SM, Miles LA, Wiley JS, Huang X, Masters CL, et al. Genomics of Alzheimer’s disease implicates the innate and adaptive immune systems. Cell Mol Life Sci. 2021;78: 7397–7426. doi: 10.1007/s00018-021-03986-5 34708251 PMC11073066

[11] Marioni RE, Harris SE, Zhang Q, McRae AF, Hagenaars SP, Hill WD, et al. GWAS on family history of Alzheimer’s disease. Transl Psychiatry. 2018;8: 99. doi: 10.1038/s41398-018-0150-6 29777097 PMC5959890

[12] Jansen IE, Savage JE, Watanabe K, Bryois J, Williams DM, Steinberg S, et al. Genome-wide meta-analysis identifies new loci and functional pathways influencing Alzheimer’s disease risk. Nat Genet. 2019;51: 404–413. doi: 10.1038/s41588-018-0311-9 30617256 PMC6836675

[13] Kunkle BW, Grenier-Boley B, Sims R, Bis JC, Damotte V, Naj AC, et al. Genetic meta-analysis of diagnosed Alzheimer’s disease identifies new risk loci and implicates Aβ, tau, immunity and lipid processing. Nat Genet. 2019;51: 414–430. doi: 10.1038/s41588-019-0358-2 30820047 PMC6463297

[14] Ninkov A, Frank JR, Maggio LA. Bibliometrics: Methods for studying academic publishing. Perspect Med Educ. 2022;11: 173–176. doi: 10.1007/s40037-021-00695-4 34914027 PMC9240160

[15] van Eck NJ, Waltman L. Software survey: VOSviewer, a computer program for bibliometric mapping. Scientometrics. 2010;84: 523–538. doi: 10.1007/s11192-009-0146-3 20585380 PMC2883932

[16] Chen C, Hu Z, Liu S, Tseng H. Emerging trends in regenerative medicine: a scientometric analysis in CiteSpace. Expert Opin Biol Ther. 2012;12: 593–608. doi: 10.1517/14712598.2012.674507 22443895

[17] Lei K, Wang X, Liu Y, Sun T, Xie W. Global research hotspots and trends of the Notch signaling pathway in the field of cancer: a bibliometric study. Am J Transl Res. 2022;14: 4918–4930. 35958476 PMC9360898

[18] Scheiblich H, Trombly M, Ramirez A, Heneka MT. Neuroimmune Connections in Aging and Neurodegenerative Diseases. Trends Immunol. 2020;41: 300–312. doi: 10.1016/j.it.2020.02.002 32147113

[19] Roses A, Sundseth S, Saunders A, Gottschalk W, Burns D, Lutz M. Understanding the genetics of APOE and TOMM40 and role of mitochondrial structure and function in clinical pharmacology of Alzheimer’s disease. Alzheimers Dement. 2016;12: 687–694. doi: 10.1016/j.jalz.2016.03.015 27154058

[20] Wolfe CM, Fitz NF, Nam KN, Lefterov I, Koldamova R. The Role of APOE and TREM2 in Alzheimer’s Disease-Current Understanding and Perspectives. Int J Mol Sci. 2018;20: 81. doi: 10.3390/ijms20010081 30587772 PMC6337314

[21] He Z, Liu L, Wang C, Le Guen Y, Lee J, Gogarten S, et al. Identification of putative causal loci in whole-genome sequencing data via knockoff statistics. Nat Commun. 2021;12: 3152. doi: 10.1038/s41467-021-22889-4 34035245 PMC8149672

[22] Serrano-Pozo A, Das S, Hyman BT. APOE and Alzheimer’s disease: advances in genetics, pathophysiology, and therapeutic approaches. Lancet Neurol. 2021;20: 68–80. doi: 10.1016/S1474-4422(20)30412-9 33340485 PMC8096522

[23] Bevan MJ. Immunology: remembrance of things past. Nature. 2002;420: 748–749. doi: 10.1038/420748a 12490927

[24] Long JM, Holtzman DM. Alzheimer Disease: An Update on Pathobiology and Treatment Strategies. Cell. 2019;179: 312–339. doi: 10.1016/j.cell.2019.09.001 31564456 PMC6778042

[25] Gate D, Saligrama N, Leventhal O, Yang AC, Unger MS, Middeldorp J, et al. Clonally expanded CD8 T cells patrol the cerebrospinal fluid in Alzheimer’s disease. Nature. 2020;577: 399–404. doi: 10.1038/s41586-019-1895-7 31915375 PMC7445078

[26] Muszyński P, Groblewska M, Kulczyńska-Przybik A, Kułakowska A, Mroczko B. YKL-40 as a Potential Biomarker and a Possible Target in Therapeutic Strategies of Alzheimer’s Disease. Curr Neuropharmacol. 2017;15: 906–917. doi: 10.2174/1570159X15666170208124324 28183245 PMC5652033

[27] Chaudhari K, Wong JM, Vann PH, Como T, O’Bryant SE, Sumien N. ApoE Genotype-Dependent Response to Antioxidant and Exercise Interventions on Brain Function. Antioxidants (Basel). 2020;9: 553. doi: 10.3390/antiox9060553 32630431 PMC7346214

[28] Palmqvist S, Janelidze S, Quiroz YT, Zetterberg H, Lopera F, Stomrud E, et al. Discriminative Accuracy of Plasma Phospho-tau217 for Alzheimer Disease vs Other Neurodegenerative Disorders. JAMA. 2020;324: 772–781. doi: 10.1001/jama.2020.12134 32722745 PMC7388060

[29] Gonzalez MC, Ashton NJ, Gomes BF, Tovar-Rios DA, Blanc F, Karikari TK, et al. Association of Plasma p-tau181 and p-tau231 Concentrations With Cognitive Decline in Patients With Probable Dementia With Lewy Bodies. JAMA Neurol. 2022;79: 32–37. doi: 10.1001/jamaneurol.2021.4222 34807233 PMC8609462

[30] Z H, P Jr, D Pm, L G, H W. Data-driven causal model discovery and personalized prediction in Alzheimer’s disease. NPJ digital medicine. 2022;5. doi: 10.1038/s41746-022-00632-7 36076010 PMC9458727

[31] Kim S, Swaminathan S, Shen L, Risacher SL, Nho K, Foroud T, et al. Genome-wide association study of CSF biomarkers Abeta1-42, t-tau, and p-tau181p in the ADNI cohort. Neurology. 2011;76: 69–79. doi: 10.1212/WNL.0b013e318204a397 21123754 PMC3030225

[32] Ramirez A, van der Flier WM, Herold C, Ramonet D, Heilmann S, Lewczuk P, et al. SUCLG2 identified as both a determinator of CSF Aβ1–42 levels and an attenuator of cognitive decline in Alzheimer’s disease. Hum Mol Genet. 2014;23: 6644–6658. doi: 10.1093/hmg/ddu372 25027320 PMC4240204

[33] Götz J, Ittner LM. Animal models of Alzheimer’s disease and frontotemporal dementia. Nat Rev Neurosci. 2008;9: 532–544. doi: 10.1038/nrn2420 18568014

[34] Hamm V, Héraud C, Bott J-B, Herbeaux K, Strittmatter C, Mathis C, et al. Differential contribution of APP metabolites to early cognitive deficits in a TgCRND8 mouse model of Alzheimer’s disease. Sci Adv. 2017;3: e1601068. doi: 10.1126/sciadv.1601068 28275722 PMC5325539

[35] Bowden J, Holmes MV. Meta-analysis and Mendelian randomization: A review. Res Synth Methods. 2019;10: 486–496. doi: 10.1002/jrsm.1346 30861319 PMC6973275

[36] Liu Z, Qin Y, Wu T, Tubbs JD, Baum L, Mak TSH, et al. Reciprocal causation mixture model for robust Mendelian randomization analysis using genome-scale summary data. Nat Commun. 2023;14: 1131. doi: 10.1038/s41467-023-36490-4 36854672 PMC9975185

[37] Multicenter Acute Stroke Trial—Europe Study Group, Hommel M, Cornu C, Boutitie F, Boissel JP. Thrombolytic therapy with streptokinase in acute ischemic stroke. N Engl J Med. 1996;335: 145–150. doi: 10.1056/NEJM199607183350301 8657211

[38] Urnov FD, Miller JC, Lee Y-L, Beausejour CM, Rock JM, Augustus S, et al. Highly efficient endogenous human gene correction using designed zinc-finger nucleases. Nature. 2005;435: 646–651. doi: 10.1038/nature03556 15806097

[39] Ertekin-Taner N. Genetics of Alzheimer disease in the pre- and post-GWAS era. Alzheimers Res Ther. 2010;2: 3. doi: 10.1186/alzrt26 20236449 PMC2874262

[40] Lambert JC, Ibrahim-Verbaas CA, Harold D, Naj AC, Sims R, Bellenguez C, et al. Meta-analysis of 74,046 individuals identifies 11 new susceptibility loci for Alzheimer’s disease. Nat Genet. 2013;45: 1452–1458. doi: 10.1038/ng.2802 24162737 PMC3896259

[41] Yang Z, Wang C, Liu L, Khan A, Lee A, Vardarajan B, et al. CARMA is a new Bayesian model for fine-mapping in genome-wide association meta-analyses. Nat Genet. 2023;55: 1057–1065. doi: 10.1038/s41588-023-01392-0 37169873

[42] Chakraborty D, Zhuang Z, Xue H, Fiecas MB, Shen X, Pan W, et al. Deep Learning-Based Feature Extraction with MRI Data in Neuroimaging Genetics for Alzheimer’s Disease. Genes (Basel). 2023;14: 626. doi: 10.3390/genes14030626 36980898 PMC10047952

[43] Alatrany AS, Hussain AJ, Mustafina J, Al-Jumeily D. Machine Learning Approaches and Applications in Genome Wide Association Study for Alzheimer’s Disease: A Systematic Review. IEEE ACCESS. 2022; 62831–62847. doi: 10.1109/ACCESS.2022.3182543

[44] Bruni AC, Bernardi L, Gabelli C. From beta amyloid to altered proteostasis in Alzheimer’s disease. Ageing Res Rev. 2020;64: 101126. doi: 10.1016/j.arr.2020.101126 32683041

[45] Mahley RW, Rall SC. Apolipoprotein E: far more than a lipid transport protein. Annu Rev Genomics Hum Genet. 2000;1: 507–537. doi: 10.1146/annurev.genom.1.1.507 11701639

[46] Liu C-C, Liu C-C, Kanekiyo T, Xu H, Bu G. Apolipoprotein E and Alzheimer disease: risk, mechanisms and therapy. Nat Rev Neurol. 2013;9: 106–118. doi: 10.1038/nrneurol.2012.263 23296339 PMC3726719

[47] Strittmatter WJ, Saunders AM, Goedert M, Weisgraber KH, Dong LM, Jakes R, et al. Isoform-specific interactions of apolipoprotein E with microtubule-associated protein tau: implications for Alzheimer disease. Proc Natl Acad Sci U S A. 1994;91: 11183–11186. doi: 10.1073/pnas.91.23.11183 7972031 PMC45191

[48] Fleming LM, Weisgraber KH, Strittmatter WJ, Troncoso JC, Johnson GV. Differential binding of apolipoprotein E isoforms to tau and other cytoskeletal proteins. Exp Neurol. 1996;138: 252–260. doi: 10.1006/exnr.1996.0064 8620924

[49] Shi Y, Yamada K, Liddelow SA, Smith ST, Zhao L, Luo W, et al. ApoE4 markedly exacerbates tau-mediated neurodegeneration in a mouse model of tauopathy. Nature. 2017;549: 523–527. doi: 10.1038/nature24016 28959956 PMC5641217

[50] Tcw J, Qian L, Pipalia NH, Chao MJ, Liang SA, Shi Y, et al. Cholesterol and matrisome pathways dysregulated in astrocytes and microglia. Cell. 2022;185: 2213–2233.e25. doi: 10.1016/j.cell.2022.05.017 35750033 PMC9340815

[51] Mok KK-S, Yeung SH-S, Cheng GW-Y, Ma IW-T, Lee RH-S, Herrup K, et al. Apolipoprotein E ε4 disrupts oligodendrocyte differentiation by interfering with astrocyte-derived lipid transport. J Neurochem. 2023;165: 55–75. doi: 10.1111/jnc.15748 36549843

[52] Calderón-Garcidueñas L, Hernández-Luna J, Aiello-Mora M, Brito-Aguilar R, Evelson PA, Villarreal-Ríos R, et al. APOE Peripheral and Brain Impact: APOE4 Carriers Accelerate Their Alzheimer Continuum and Have a High Risk of Suicide in PM2.5 Polluted Cities. Biomolecules. 2023;13: 927. doi: 10.3390/biom13060927 37371506 PMC10296707

[53] Chan ML, Meyer OL, Farias ST, Whitmer RA, Rajan K, Olichney J, et al. APOE Effects on Late Life Cognitive Trajectories in Diverse Racial/Ethnic Groups. J Int Neuropsychol Soc. 2023;29: 126–135. doi: 10.1017/S1355617722000030 35243969 PMC9440953

[54] Llibre-Guerra JJ, Li J, Qian Y, Llibre-Rodriguez J de J, Jiménez-Velázquez IZ, Acosta D, et al. Apolipoprotein E (APOE) genotype, dementia, and memory performance among Caribbean Hispanic versus US populations. Alzheimers Dement. 2023;19: 602–610. doi: 10.1002/alz.12699 35661582 PMC9719569

[55] Walters S, Contreras AG, Eissman JM, Mukherjee S, Lee ML, Choi S-E, et al. Associations of Sex, Race, and Apolipoprotein E Alleles With Multiple Domains of Cognition Among Older Adults. JAMA Neurol. 2023;80: 929–939. doi: 10.1001/jamaneurol.2023.2169 37459083 PMC10352930

[56] Altmann A, Tian L, Henderson VW, Greicius MD, Alzheimer’s Disease Neuroimaging Initiative Investigators. Sex modifies the APOE-related risk of developing Alzheimer disease. Ann Neurol. 2014;75: 563–573. doi: 10.1002/ana.24135 24623176 PMC4117990

[57] Beydoun MA, Boueiz A, Abougergi MS, Kitner-Triolo MH, Beydoun HA, Resnick SM, et al. Sex differences in the association of the apolipoprotein E epsilon 4 allele with incidence of dementia, cognitive impairment, and decline. Neurobiol Aging. 2012;33: 720–731.e4. doi: 10.1016/j.neurobiolaging.2010.05.017 20619505 PMC2974952

[58] Hohman TJ, Dumitrescu L, Barnes LL, Thambisetty M, Beecham G, Kunkle B, et al. Sex-Specific Association of Apolipoprotein E With Cerebrospinal Fluid Levels of Tau. JAMA Neurol. 2018;75: 989–998. doi: 10.1001/jamaneurol.2018.0821 29801024 PMC6142927

[59] Damoiseaux JS, Seeley WW, Zhou J, Shirer WR, Coppola G, Karydas A, et al. Gender modulates the APOE ε4 effect in healthy older adults: convergent evidence from functional brain connectivity and spinal fluid tau levels. J Neurosci. 2012;32: 8254–8262. doi: 10.1523/JNEUROSCI.0305-12.2012 22699906 PMC3394933

[60] Jenne DE, Tschopp J. Clusterin: the intriguing guises of a widely expressed glycoprotein. Trends Biochem Sci. 1992;17: 154–159. doi: 10.1016/0968-0004(92)90325-4 1585460

[61] DeMattos RB, Brendza RP, Heuser JE, Kierson M, Cirrito JR, Fryer J, et al. Purification and characterization of astrocyte-secreted apolipoprotein E and J-containing lipoproteins from wild-type and human apoE transgenic mice. Neurochem Int. 2001;39: 415–425. doi: 10.1016/s0197-0186(01)00049-3 11578777

[62] DeMattos RB, Cirrito JR, Parsadanian M, May PC, O’Dell MA, Taylor JW, et al. ApoE and clusterin cooperatively suppress Abeta levels and deposition: evidence that ApoE regulates extracellular Abeta metabolism in vivo. Neuron. 2004;41: 193–202. doi: 10.1016/s0896-6273(03)00850-x 14741101

[63] Bell RD, Sagare AP, Friedman AE, Bedi GS, Holtzman DM, Deane R, et al. Transport pathways for clearance of human Alzheimer’s amyloid beta-peptide and apolipoproteins E and J in the mouse central nervous system. J Cereb Blood Flow Metab. 2007;27: 909–918. doi: 10.1038/sj.jcbfm.9600419 17077814 PMC2853021

[64] Chen LH, Kao PYP, Fan YH, Ho DTY, Chan CSY, Yik PY, et al. Polymorphisms of CR1, CLU and PICALM confer susceptibility of Alzheimer’s disease in a southern Chinese population. Neurobiol Aging. 2012;33: 210.e1–7. doi: 10.1016/j.neurobiolaging.2011.09.016 22015308

[65] Wang H-Z, Bi R, Hu Q-X, Xiang Q, Zhang C, Zhang D-F, et al. Validating GWAS-Identified Risk Loci for Alzheimer’s Disease in Han Chinese Populations. Mol Neurobiol. 2016;53: 379–390. doi: 10.1007/s12035-014-9015-z 25452228

[66] Jiao B, Liu X, Zhou L, Wang MH, Zhou Y, Xiao T, et al. Polygenic Analysis of Late-Onset Alzheimer’s Disease from Mainland China. PLoS One. 2015;10: e0144898. doi: 10.1371/journal.pone.0144898 26680604 PMC4683047

[67] Huang F, Shang Y, Luo Y, Wu P, Huang X, Tan X, et al. Lower Prevalence of Alzheimer’s Disease among Tibetans: Association with Religious and Genetic Factors. J Alzheimers Dis. 2016;50: 659–667. doi: 10.3233/JAD-150697 26757186

[68] Mengel-From J, Christensen K, McGue M, Christiansen L. Genetic variations in the CLU and PICALM genes are associated with cognitive function in the oldest old. Neurobiol Aging. 2011;32: 554.e7–11. doi: 10.1016/j.neurobiolaging.2010.07.016 20739100 PMC3042035

[69] Mengel-From J, Thinggaard M, Lindahl-Jacobsen R, McGue M, Christensen K, Christiansen L. CLU genetic variants and cognitive decline among elderly and oldest old. PLoS One. 2013;8: e79105. doi: 10.1371/journal.pone.0079105 24244428 PMC3828341

[70] Du W, Tan J, Xu W, Chen J, Wang L. Association between clusterin gene polymorphism rs11136000 and late-onset Alzheimer’s disease susceptibility: A review and meta-analysis of case-control studies. Exp Ther Med. 2016;12: 2915–2927. doi: 10.3892/etm.2016.3734 27882096 PMC5103725

[71] L G, W H, L J, L J, L H, M G, et al. The CLU gene rs11136000 variant is significantly associated with Alzheimer’s disease in Caucasian and Asian populations. Neuromolecular medicine. 2014;16. doi: 10.1007/s12017-013-8250-1 23892938

[72] Zhu B, Wang RM, Wang JT, Chen RL, Zheng YF, Zhang L, et al. Correlation of rs9331888 polymorphism with Alzheimer’s disease among Caucasian and Chinese populations: a meta-analysis and systematic review. Metab Brain Dis. 2017;32: 981–989. doi: 10.1007/s11011-017-9957-8 28168383

[73] Zhang S, Li X, Ma G, Jiang Y, Liao M, Feng R, et al. CLU rs9331888 Polymorphism Contributes to Alzheimer’s Disease Susceptibility in Caucasian But Not East Asian Populations. Mol Neurobiol. 2016;53: 1446–1451. doi: 10.1007/s12035-015-9098-1 25633098

[74] Nickells M, Hauhart R, Krych M, Subramanian VB, Geoghegan-Barek K, Marsh HC, et al. Mapping epitopes for 20 monoclonal antibodies to CR1. Clin Exp Immunol. 1998;112: 27–33. doi: 10.1046/j.1365-2249.1998.00549.x 9566786 PMC1904933

[75] Brouwers N, Van Cauwenberghe C, Engelborghs S, Lambert J-C, Bettens K, Le Bastard N, et al. Alzheimer risk associated with a copy number variation in the complement receptor 1 increasing C3b/C4b binding sites. Mol Psychiatry. 2012;17: 223–233. doi: 10.1038/mp.2011.24 21403675 PMC3265835

[76] Keenan BT, Shulman JM, Chibnik LB, Raj T, Tran D, Sabuncu MR, et al. A coding variant in CR1 interacts with APOE-ε4 to influence cognitive decline. Hum Mol Genet. 2012;21: 2377–2388. doi: 10.1093/hmg/dds054 22343410 PMC3335317

[77] Kim S, Nho K, Ramanan VK, Lai D, Foroud TM, Lane K, et al. Genetic Influences on Plasma Homocysteine Levels in African Americans and Yoruba Nigerians. J Alzheimers Dis. 2016;49: 991–1003. doi: 10.3233/JAD-150651 26519441 PMC4822513

[78] Shulman JM, Imboywa S, Giagtzoglou N, Powers MP, Hu Y, Devenport D, et al. Functional screening in Drosophila identifies Alzheimer’s disease susceptibility genes and implicates Tau-mediated mechanisms. Hum Mol Genet. 2014;23: 870–877. doi: 10.1093/hmg/ddt478 24067533 PMC3900103

[79] Xue Y-Y, Chen Y-H, Lin R-R, Huang H-F, Wu Z-Y, Tao Q-Q, et al. Alzheimer’s disease susceptibility locus in CD2AP is associated with increased cerebrospinal fluid tau levels in mild cognitive impairment. Neurosci Lett. 2022;771: 136419. doi: 10.1016/j.neulet.2021.136419 34958910

[80] Shulman JM, Chen K, Keenan BT, Chibnik LB, Fleisher A, Thiyyagura P, et al. Genetic Susceptibility for Alzheimer’s Disease Neuritic Plaque Pathology. JAMA Neurol. 2013;70: 1150–1157. doi: 10.1001/jamaneurol.2013.2815 23836404 PMC3773291

[81] Liao F, Jiang H, Srivatsan S, Xiao Q, Lefton KB, Yamada K, et al. Effects of CD2-associated protein deficiency on amyloid-β in neuroblastoma cells and in an APP transgenic mouse model. Mol Neurodegener. 2015;10: 12. doi: 10.1186/s13024-015-0006-y 25887956 PMC4374406

[82] Chen H, Wu G, Jiang Y, Feng R, Liao M, Zhang L, et al. Analyzing 54,936 Samples Supports the Association Between CD2AP rs9349407 Polymorphism and Alzheimer’s Disease Susceptibility. Mol Neurobiol. 2015;52: 1–7. doi: 10.1007/s12035-014-8834-2 25092125

[83] Hollingworth P, Harold D, Sims R, Gerrish A, Lambert J-C, Carrasquillo MM, et al. Common variants at ABCA7, MS4A6A/MS4A4E, EPHA1, CD33 and CD2AP are associated with Alzheimer’s disease. Nat Genet. 2011;43: 429–435. doi: 10.1038/ng.803 21460840 PMC3084173

[84] Bertram L, Lange C, Mullin K, Parkinson M, Hsiao M, Hogan MF, et al. Genome-wide association analysis reveals putative Alzheimer’s disease susceptibility loci in addition to APOE. Am J Hum Genet. 2008;83: 623–632. doi: 10.1016/j.ajhg.2008.10.008 18976728 PMC2668052

[85] Karch CM, Jeng AT, Nowotny P, Cady J, Cruchaga C, Goate AM. Expression of novel Alzheimer’s disease risk genes in control and Alzheimer’s disease brains. PLoS One. 2012;7: e50976. doi: 10.1371/journal.pone.0050976 23226438 PMC3511432

[86] N Ac, J G, B Gw, W Ls, V Bn, B J, et al. Common variants at MS4A4/MS4A6E, CD2AP, CD33 and EPHA1 are associated with late-onset Alzheimer’s disease. Nature genetics. 2011;43. doi: 10.1038/ng.801 21460841 PMC3090745

[87] Malik M, Simpson JF, Parikh I, Wilfred BR, Fardo DW, Nelson PT, et al. CD33 Alzheimer’s risk-altering polymorphism, CD33 expression, and exon 2 splicing. J Neurosci. 2013;33: 13320–13325. doi: 10.1523/JNEUROSCI.1224-13.2013 23946390 PMC3742922

[88] Tortora F, Rendina A, Angiolillo A, Di Costanzo A, Aniello F, Donizetti A, et al. CD33 rs2455069 SNP: Correlation with Alzheimer’s Disease and Hypothesis of Functional Role. Int J Mol Sci. 2022;23: 3629. doi: 10.3390/ijms23073629 35408990 PMC8998932

[89] Bradshaw EM, Chibnik LB, Keenan BT, Ottoboni L, Raj T, Tang A, et al. CD33 Alzheimer’s disease locus: altered monocyte function and amyloid biology. Nat Neurosci. 2013;16: 848–850. doi: 10.1038/nn.3435 23708142 PMC3703870

[90] Griciuc A, Serrano-Pozo A, Parrado AR, Lesinski AN, Asselin CN, Mullin K, et al. Alzheimer’s disease risk gene CD33 inhibits microglial uptake of amyloid beta. Neuron. 2013;78: 631–643. doi: 10.1016/j.neuron.2013.04.014 23623698 PMC3706457

[91] Karch CM, Goate AM. Alzheimer’s disease risk genes and mechanisms of disease pathogenesis. Biol Psychiatry. 2015;77: 43–51. doi: 10.1016/j.biopsych.2014.05.006 24951455 PMC4234692

[92] Lai K-O, Ip NY. Synapse development and plasticity: roles of ephrin/Eph receptor signaling. Curr Opin Neurobiol. 2009;19: 275–283. doi: 10.1016/j.conb.2009.04.009 19497733

[93] Ivanov AI, Romanovsky AA. Putative dual role of ephrin-Eph receptor interactions in inflammation. IUBMB Life. 2006;58: 389–394. doi: 10.1080/15216540600756004 16801213

[94] Duffy SL, Coulthard MG, Spanevello MD, Herath NI, Yeadon TM, McCarron JK, et al. Generation and characterization of EphA1 receptor tyrosine kinase reporter knockout mice. Genesis. 2008;46: 553–561. doi: 10.1002/dvg.20434 18802966

[95] Talebi M, Delpak A, Khalaj-Kondori M, Sadigh-Eteghad S, Talebi M, Mehdizadeh E, et al. ABCA7 and EphA1 Genes Polymorphisms in Late-Onset Alzheimer’s Disease. J Mol Neurosci. 2020;70: 167–173. doi: 10.1007/s12031-019-01420-x 31659653

[96] Ando K, Brion J-P, Stygelbout V, Suain V, Authelet M, Dedecker R, et al. Clathrin adaptor CALM/PICALM is associated with neurofibrillary tangles and is cleaved in Alzheimer’s brains. Acta Neuropathol. 2013;125: 861–878. doi: 10.1007/s00401-013-1111-z 23589030

[97] Zhao Z, Sagare AP, Ma Q, Halliday MR, Kong P, Kisler K, et al. Central role for PICALM in amyloid-β blood-brain barrier transcytosis and clearance. Nat Neurosci. 2015;18: 978–987. doi: 10.1038/nn.4025 26005850 PMC4482781

[98] Xu W, Tan C-C, Cao X-P, Tan L, Alzheimer’s Disease Neuroimaging Initiative. Association of Alzheimer’s disease risk variants on the PICALM gene with PICALM expression, core biomarkers, and feature neurodegeneration. Aging (Albany NY). 2020;12: 21202–21219. doi: 10.18632/aging.103814 33170153 PMC7695360

[99] Vasquez JB, Fardo DW, Estus S. ABCA7 expression is associated with Alzheimer’s disease polymorphism and disease status. Neurosci Lett. 2013;556: 58–62. doi: 10.1016/j.neulet.2013.09.058 24141082 PMC3863933

[100] Jehle AW, Gardai SJ, Li S, Linsel-Nitschke P, Morimoto K, Janssen WJ, et al. ATP-binding cassette transporter A7 enhances phagocytosis of apoptotic cells and associated ERK signaling in macrophages. J Cell Biol. 2006;174: 547–556. doi: 10.1083/jcb.200601030 16908670 PMC2064260

[101] Dib S, Pahnke J, Gosselet F. Role of ABCA7 in Human Health and in Alzheimer’s Disease. Int J Mol Sci. 2021;22: 4603. doi: 10.3390/ijms22094603 33925691 PMC8124837

[102] Wang N, Lan D, Gerbod-Giannone M, Linsel-Nitschke P, Jehle AW, Chen W, et al. ATP-binding cassette transporter A7 (ABCA7) binds apolipoprotein A-I and mediates cellular phospholipid but not cholesterol efflux. J Biol Chem. 2003;278: 42906–42912. doi: 10.1074/jbc.M307831200 12917409

[103] Abe-Dohmae S, Ikeda Y, Matsuo M, Hayashi M, Okuhira K, Ueda K, et al. Human ABCA7 supports apolipoprotein-mediated release of cellular cholesterol and phospholipid to generate high density lipoprotein. J Biol Chem. 2004;279: 604–611. doi: 10.1074/jbc.M309888200 14570867

[104] Kim WS, Li H, Ruberu K, Chan S, Elliott DA, Low JK, et al. Deletion of Abca7 Increases Cerebral Amyloid-β Accumulation in the J20 Mouse Model of Alzheimer’s Disease. J Neurosci. 2013;33: 4387–4394. doi: 10.1523/JNEUROSCI.4165-12.2013 23467355 PMC6704948

[105] Stepler KE, Gillyard TR, Reed CB, Avery TM, Davis JS, Robinson RAS. ABCA7, a Genetic Risk Factor Associated with Alzheimer’s Disease Risk in African Americans. J Alzheimers Dis. 2022;86: 5–19. doi: 10.3233/JAD-215306 35034901 PMC10984370

[106] Jiao B, Xiao X, Yuan Z, Guo L, Liao X, Zhou Y, et al. Associations of risk genes with onset age and plasma biomarkers of Alzheimer’s disease: a large case-control study in mainland China. Neuropsychopharmacology. 2022;47: 1121–1127. doi: 10.1038/s41386-021-01258-1 35001095 PMC8938514

[107] Carrasquillo MM, Crook JE, Pedraza O, Thomas CS, Pankratz VS, Allen M, et al. Late-onset Alzheimer’s risk variants in memory decline, incident mild cognitive impairment, and Alzheimer’s disease. Neurobiol Aging. 2015;36: 60–67. doi: 10.1016/j.neurobiolaging.2014.07.042 25189118 PMC4268433

[108] Nettiksimmons J, Tranah G, Evans DS, Yokoyama JS, Yaffe K. Gene-based aggregate SNP associations between candidate AD genes and cognitive decline. Age (Dordr). 2016;38: 41. doi: 10.1007/s11357-016-9885-2 27005436 PMC5005889

[109] Sessa G, Podini P, Mariani M, Meroni A, Spreafico R, Sinigaglia F, et al. Distribution and signaling of TREM2/DAP12, the receptor system mutated in human polycystic lipomembraneous osteodysplasia with sclerosing leukoencephalopathy dementia. Eur J Neurosci. 2004;20: 2617–2628. doi: 10.1111/j.1460-9568.2004.03729.x 15548205

[110] Guerreiro R, Wojtas A, Bras J, Carrasquillo M, Rogaeva E, Majounie E, et al. TREM2 variants in Alzheimer’s disease. N Engl J Med. 2013;368: 117–127. doi: 10.1056/NEJMoa1211851 23150934 PMC3631573

[111] Jonsson T, Stefansson H, Steinberg S, Jonsdottir I, Jonsson PV, Snaedal J, et al. Variant of TREM2 associated with the risk of Alzheimer’s disease. N Engl J Med. 2013;368: 107–116. doi: 10.1056/NEJMoa1211103 23150908 PMC3677583

[112] Coon KD, Myers AJ, Craig DW, Webster JA, Pearson JV, Lince DH, et al. A high-density whole-genome association study reveals that APOE is the major susceptibility gene for sporadic late-onset Alzheimer’s disease. J Clin Psychiatry. 2007;68: 613–618. doi: 10.4088/jcp.v68n0419 17474819

[113] Abraham R, Moskvina V, Sims R, Hollingworth P, Morgan A, Georgieva L, et al. A genome-wide association study for late-onset Alzheimer’s disease using DNA pooling. BMC Med Genomics. 2008;1: 44. doi: 10.1186/1755-8794-1-44 18823527 PMC2570675

[114] Potkin SG, Guffanti G, Lakatos A, Turner JA, Kruggel F, Fallon JH, et al. Hippocampal atrophy as a quantitative trait in a genome-wide association study identifying novel susceptibility genes for Alzheimer’s disease. PLoS One. 2009;4: e6501. doi: 10.1371/journal.pone.0006501 19668339 PMC2719581

[115] Seshadri S, Fitzpatrick AL, Ikram MA, DeStefano AL, Gudnason V, Boada M, et al. Genome-wide analysis of genetic loci associated with Alzheimer disease. JAMA. 2010;303: 1832–1840. doi: 10.1001/jama.2010.574 20460622 PMC2989531

[116] Hu X, Pickering E, Liu YC, Hall S, Fournier H, Katz E, et al. Meta-analysis for genome-wide association study identifies multiple variants at the BIN1 locus associated with late-onset Alzheimer’s disease. PLoS One. 2011;6: e16616. doi: 10.1371/journal.pone.0016616 21390209 PMC3044719

[117] Liu C, Yu J. Genome-Wide Association Studies for Cerebrospinal Fluid Soluble TREM2 in Alzheimer’s Disease. Frontiers in Aging Neuroscience. 2019;11. doi: 10.3389/fnagi.2019.00297 31708768 PMC6823606

[118] Afsar A, Chacon Castro MDC, Soladogun AS, Zhang L. Recent Development in the Understanding of Molecular and Cellular Mechanisms Underlying the Etiopathogenesis of Alzheimer’s Disease. Int J Mol Sci. 2023;24: 7258. doi: 10.3390/ijms24087258 37108421 PMC10138573

[119] Deane R, Sagare A, Hamm K, Parisi M, Lane S, Finn MB, et al. apoE isoform-specific disruption of amyloid beta peptide clearance from mouse brain. J Clin Invest. 2008;118: 4002–4013. doi: 10.1172/JCI36663 19033669 PMC2582453

[120] Basak JM, Kim J, Pyatkivskyy Y, Wildsmith KR, Jiang H, Parsadanian M, et al. Measurement of apolipoprotein E and amyloid β clearance rates in the mouse brain using bolus stable isotope labeling. Mol Neurodegener. 2012;7: 14. doi: 10.1186/1750-1326-7-14 22512932 PMC3405485

[121] Qi X-M, Ma J-F. The role of amyloid beta clearance in cerebral amyloid angiopathy: more potential therapeutic targets. Transl Neurodegener. 2017;6: 22. doi: 10.1186/s40035-017-0091-7 28824801 PMC5559841

[122] Gold CA, Budson AE. Memory loss in Alzheimer’s disease: implications for development of therapeutics. Expert Rev Neurother. 2008;8: 1879–1891. doi: 10.1586/14737175.8.12.1879 19086882 PMC2655107

[123] Selkoe DJ, Hardy J. The amyloid hypothesis of Alzheimer’s disease at 25 years. EMBO Mol Med. 2016;8: 595–608. doi: 10.15252/emmm.201606210 27025652 PMC4888851

[124] Parikh V, Bernard CS, Naughton SX, Yegla B. Interactions between Aβ oligomers and presynaptic cholinergic signaling: age-dependent effects on attentional capacities. Behav Brain Res. 2014;274: 30–42. doi: 10.1016/j.bbr.2014.07.046 25101540 PMC4179990

[125] Kong F, Wu T, Dai J, Zhai Z, Cai J, Zhu Z, et al. Glucagon-like peptide 1 (GLP-1) receptor agonists in experimental Alzheimer’s disease models: a systematic review and meta-analysis of preclinical studies. Front Pharmacol. 2023;14: 1205207. doi: 10.3389/fphar.2023.1205207 37771725 PMC10525376

[126] Yu L, Chibnik LB, Srivastava GP, Pochet N, Yang J, Xu J, et al. Association of Brain DNA methylation in SORL1, ABCA7, HLA-DRB5, SLC24A4, and BIN1 with pathological diagnosis of Alzheimer disease. JAMA Neurol. 2015;72: 15–24. doi: 10.1001/jamaneurol.2014.3049 25365775 PMC4344367

[127] Zhu X-C, Liu L, Dai W-Z, Ma T. Crry silencing alleviates Alzheimer’s disease injury by regulating neuroinflammatory cytokines and the complement system. Neural Regen Res. 2022;17: 1841–1849. doi: 10.4103/1673-5374.332160 35017447 PMC8820699

[128] Chakrabarti S, Khemka VK, Banerjee A, Chatterjee G, Ganguly A, Biswas A. Metabolic Risk Factors of Sporadic Alzheimer’s Disease: Implications in the Pathology, Pathogenesis and Treatment. Aging Dis. 2015;6: 282–299. doi: 10.14336/AD.2014.002 26236550 PMC4509477

[129] Tai LM, Ghura S, Koster KP, Liakaite V, Maienschein-Cline M, Kanabar P, et al. APOE-modulated Aβ-induced neuroinflammation in Alzheimer’s disease: current landscape, novel data, and future perspective. J Neurochem. 2015;133: 465–488. doi: 10.1111/jnc.13072 25689586 PMC4400246

[130] Peng Y, Gao P, Shi L, Chen L, Liu J, Long J. Central and Peripheral Metabolic Defects Contribute to the Pathogenesis of Alzheimer’s Disease: Targeting Mitochondria for Diagnosis and Prevention. Antioxid Redox Signal. 2020;32: 1188–1236. doi: 10.1089/ars.2019.7763 32050773 PMC7196371

[131] Zhang Z-G, Li Y, Ng CT, Song Y-Q. Inflammation in Alzheimer’s Disease and Molecular Genetics: Recent Update. Arch Immunol Ther Exp (Warsz). 2015;63: 333–344. doi: 10.1007/s00005-015-0351-0 26232392

[132] Cao D, Shu X, Zhu D, Liang S, Hasan M, Gong S. Lipid-coated ZnO nanoparticles synthesis, characterization and cytotoxicity studies in cancer cell. Nano Converg. 2020;7: 14. doi: 10.1186/s40580-020-00224-9 32328852 PMC7181468

[133] Schultz SA, Boots EA, Darst BF, Zetterberg H, Blennow K, Edwards DF, et al. Cardiorespiratory fitness alters the influence of a polygenic risk score on biomarkers of AD. Neurology. 2017;88: 1650–1658. doi: 10.1212/WNL.0000000000003862 28341646 PMC5405766

[134] Barbero-Camps E, Roca-Agujetas V, Bartolessis I, de Dios C, Fernández-Checa JC, Marí M, et al. Cholesterol impairs autophagy-mediated clearance of amyloid beta while promoting its secretion. Autophagy. 2018;14: 1129–1154. doi: 10.1080/15548627.2018.1438807 29862881 PMC6103708

[135] Paquet C, Nicoll JA, Love S, Mouton-Liger F, Holmes C, Hugon J, et al. Downregulated apoptosis and autophagy after anti-Aβ immunotherapy in Alzheimer’s disease. Brain Pathol. 2018;28: 603–610. doi: 10.1111/bpa.12567 29027727 PMC8028546

[136] Morley JE, Farr SA, Nguyen AD. Alzheimer Disease. Clin Geriatr Med. 2018;34: 591–601. doi: 10.1016/j.cger.2018.06.006 30336989

[137] Zhao J, Fu Y, Yamazaki Y, Ren Y, Davis MD, Liu C-C, et al. APOE4 exacerbates synapse loss and neurodegeneration in Alzheimer’s disease patient iPSC-derived cerebral organoids. Nat Commun. 2020;11: 5540. doi: 10.1038/s41467-020-19264-0 33139712 PMC7608683

[138] Zhao F, Zhao L, Zhou Y, Tan X, Yang Y, Ni W, et al. A Multifunctional (-)-Meptazinol-Serotonin Hybrid Ameliorates Oxidative Stress-Associated Apoptotic Neuronal Death and Memory Deficits via Activating the Nrf2/Antioxidant Enzyme Pathway. Oxid Med Cell Longev. 2023;2023: 6935947. doi: 10.1155/2023/6935947 36819782 PMC9935814

[139] Mamun AA, Uddin MS, Bin Bashar MF, Zaman S, Begum Y, Bulbul IJ, et al. Molecular Insight into the Therapeutic Promise of Targeting APOE4 for Alzheimer’s Disease. Oxid Med Cell Longev. 2020;2020: 5086250. doi: 10.1155/2020/5086250 32509144 PMC7245681

[140] Jun H-O, Kim D, Lee S-W, Lee HS, Seo JH, Kim JH, et al. Clusterin protects H9c2 cardiomyocytes from oxidative stress-induced apoptosis via Akt/GSK-3β signaling pathway. Exp Mol Med. 2011;43: 53–61. doi: 10.3858/emm.2011.43.1.006 21270507 PMC3041938

[141] Wang C, Zeng Z, Liu Q, Zhang R, Ni J. Se-methylselenocysteine inhibits apoptosis induced by clusterin knockdown in neuroblastoma N2a and SH-SY5Y cell lines. Int J Mol Sci. 2014;15: 21331–21347. doi: 10.3390/ijms151121331 25411798 PMC4264228

[142] Reitz C, Brayne C, Mayeux R. Epidemiology of Alzheimer disease. Nat Rev Neurol. 2011;7: 137–152. doi: 10.1038/nrneurol.2011.2 21304480 PMC3339565

[143] Satarug S, Garrett SH, Sens MA, Sens DA. Cadmium, environmental exposure, and health outcomes. Environ Health Perspect. 2010;118: 182–190. doi: 10.1289/ehp.0901234 20123617 PMC2831915

[144] Chen L, Liu L, Huang S. Cadmium activates the mitogen-activated protein kinase (MAPK) pathway via induction of reactive oxygen species and inhibition of protein phosphatases 2A and 5. Free Radic Biol Med. 2008;45: 1035–1044. doi: 10.1016/j.freeradbiomed.2008.07.011 18703135

[145] Y Y, Z Y, Z S, C J, Y J, W T, et al. Cadmium-induced apoptosis in neuronal cells is mediated by Fas/FasL-mediated mitochondrial apoptotic signaling pathway. Scientific reports. 2018;8. doi: 10.1038/s41598-018-27106-9 29891925 PMC5995901

[146] Zhang L, Wang H, Abel GM, Storm DR, Xia Z. The Effects of Gene-Environment Interactions Between Cadmium Exposure and Apolipoprotein E4 on Memory in a Mouse Model of Alzheimer’s Disease. Toxicol Sci. 2020;173: 189–201. doi: 10.1093/toxsci/kfz218 31626305 PMC8204948

[147] Engstrom AK, Snyder JM, Maeda N, Xia Z. Gene-environment interaction between lead and Apolipoprotein E4 causes cognitive behavior deficits in mice. Mol Neurodegener. 2017;12: 14. doi: 10.1186/s13024-017-0155-2 28173832 PMC5297175

[148] BurgenerSC, BuettnerL, Coen BuckwalterK, BeattieE, BossenAL, FickDM, et al. Evidence supporting nutritional interventions for persons in early stage Alzheimer’s disease (AD). J Nutr Health Aging. 2008;12: 18–21. doi: 10.1007/BF02982159 18165840

[149] Barberger-Gateau P, Raffaitin C, Letenneur L, Berr C, Tzourio C, Dartigues JF, et al. Dietary patterns and risk of dementia: the Three-City cohort study. Neurology. 2007;69: 1921–1930. doi: 10.1212/01.wnl.0000278116.37320.52 17998483

[150] Santibáñez M, Bolumar F, García AM. Occupational risk factors in Alzheimer’s disease: a review assessing the quality of published epidemiological studies. Occup Environ Med. 2007;64: 723–732. doi: 10.1136/oem.2006.028209 17525096 PMC2078415

[151] Sinem F, Dildar K, Gökhan E, Melda B, Orhan Y, Filiz M. The serum protein and lipid oxidation marker levels in Alzheimer’s disease and effects of cholinesterase inhibitors and antipsychotic drugs therapy. Curr Alzheimer Res. 2010;7: 463–469. doi: 10.2174/156720510791383822 20043811

[152] Frisoni GB, Boccardi M, Barkhof F, Blennow K, Cappa S, Chiotis K, et al. Strategic roadmap for an early diagnosis of Alzheimer’s disease based on biomarkers. Lancet Neurol. 2017;16: 661–676. doi: 10.1016/S1474-4422(17)30159-X 28721928

[153] Jin C, Li W, Yuan J, Xu W, Cheng Z. Association of the CR1 polymorphism with late-onset Alzheimer’s disease in Chinese Han populations: a meta-analysis. Neurosci Lett. 2012;527: 46–49. doi: 10.1016/j.neulet.2012.08.032 22960360

[154] Shen N, Chen B, Jiang Y, Feng R, Liao M, Zhang L, et al. An Updated Analysis with 85,939 Samples Confirms the Association Between CR1 rs6656401 Polymorphism and Alzheimer’s Disease. Mol Neurobiol. 2015;51: 1017–1023. doi: 10.1007/s12035-014-8761-2 24878768

